# A novel solution configuration on liquid-based endometrial cytology

**DOI:** 10.1371/journal.pone.0190851

**Published:** 2018-02-05

**Authors:** Shulan Lv, Rui Wang, Qi Wang, Lu Han, Xiaoqian Tuo, Huilian Hou, Yu Liu, Zan Shi, Qing Wang, Yan Li, Chao Sun, Xue Xue, Qiling Li

**Affiliations:** 1 Department of Obstetrics and Gynecology, First Affiliated Hospital of Xi’an Jiaotong University, Xi’an, Shaanxi, China; 2 ART Center, Northwest Women’s and Children’s Hospital, Xi’an, Shaanxi, China; 3 Department of Pathology, First Affiliated Hospital of Xi’an Jiaotong University, Xi’an, Shaanxi, China; 4 Center of Big Data and Bioinformatics, First Affiliated Hospital of Xi’an Jiaotong University, Xi’an, Shaanxi, China; Morehouse School of Medicine, UNITED STATES

## Abstract

**Objective:**

Early detection and diagnosis of endometrial carcinoma and precancerous change would undoubtedly become the most alluring part for researchers. With the emergence of endometrial brush samplers, a new upsurge in endometrial cytology is in the making. But endometrial specimens obtained by the endometrial brush samplers require special preservation solution. The objective of this study is to develop a new kind of endometrial-cell preservation solution and to test the availability compared with a patented liquid-based cell preservation solution.

**Methods:**

In this controlled study, we had 5 endometrial cases collected with Li Brush from the First Affiliated Hospital of Xi'an Jiaotong University (09/2016 to 12/2016). The samples of each case were collected 2 times separately and perserved in different perservation solutions. One was a kind of novel endometrial cell preservation solution and the other was a kind of patented liquid-based cell (LBC) preservation solution. The endometrial cells were smeared on slides by using the ZP-C automated slide preparation system and stained with Papanicolaou stain. A semi-quantitative scoring system was used to analyze the quality of slides. Statistical analysis was performed using the Wilcoxon signed rank test on the SPSS program (SPSS 18.0). In all LBC preparations, endometrial cells from the novel endometrial cells preservation solution had more cell quantity, less red blood cell fragments, and the background was cleaner compared with control group. Although the novel endometrial-cell preservation solution showed cellularity and absence of blood and debris expressed by no statistically significant differences (p = 0.063 and 0.102 respectively). The preservation period of the two kinds of liquids was equivalent.

**Conclusions:**

The novel endometrial-cell preservation solution is superior to the liquid-base cell preservation solution for cervical cells, with clear background, diagnostic cells and low cost.

## Introduction

Endometrial carcinoma (EC) is a common malignant tumor of the female genital tract in gynecology. In recent years, the incidence of EC has increased to the fourth most common cancer in women [1]. Early detection and diagnosis of EC and precancerous change would undoubtedly become the most alluring part for researchers. Endometrial biopsy, dilation and curettage (D&C) and hysteroscopy are invasive, painful and expensive. Although transvaginal ultrasound is simple and convenient, it cannot solely be used to rule out malignant endometrial lesions due to poor specificity [2]. Simple, convenient, painless endometrial collectors are used and becoming a screening tool in recent years, such as Li Brush [3], SAP-1[4] and Tao Brush [5], which can collect endometrial cells.

As we know, liquid-based cytology (LBC) has improved the quality of preparations compared with the conventional Pap smears in screening of cervical cancer [6–9].The selection of different cell preservation solution for different samples is particularly important to arrive at good microscopic images. With the emergence of endometrial brush samplers, a new upsurge in endometrial cytology is in the making. Endometrial specimens obtained by the endometrial brush samplers are preserved in liquid-based cell preservation solution nowadays, which are commonly same as preservations solution for cervical cytology specimens [10–12].

In theory, due to different cell types (squamous epithelial cells in cervix and columnar epithelial cells in endometrium) and different red-blood cell amount in the samples, the preservation solution should be different. In order to help the pathologists identify abnormal cells and improve diagnostic accuracy, we prepared a novel endometrial cell preservation solution added proper proportion of ammonium chloride based on a patented liquid-based cell preservation solution (Patent number: CN103583508-A). The purpose of this study is to validate the availability of the novel endometrial cell preservation solution.

## Materials and methods

### Instruments and reagents

Liquid-based Cytology ThinPrep System (ZP-C automatic liquid-based thin-layer cell maker, Xiaogan Hongxiang biological medical technology Co. Ltd, LCT3000), endometrial brush (Li Brush, Xi'an Meijiajia Medical Technology Co. Ltd., 20152660054); ammonium chloride (Tianjin Hengxing Chemical Reagent Manufacturing Co. Ltd. 20100408), sodium citrate (Tianjin Baishi Chemical Co. Ltd. 20090318), formaldehyde (Tianjin Tianli Chemical Reagent Co. Ltd., 20130508), sodium chloride (Shanghai Experiment Reagent Co. Ltd., 20150207), glacial acetic acid (Guangdong Project Technology Research Exploration Center, 20131017), ethanol (Xi'an Chemical Reagent Factory, 150902).

### Ethics statement and patients

**Fig 1:**
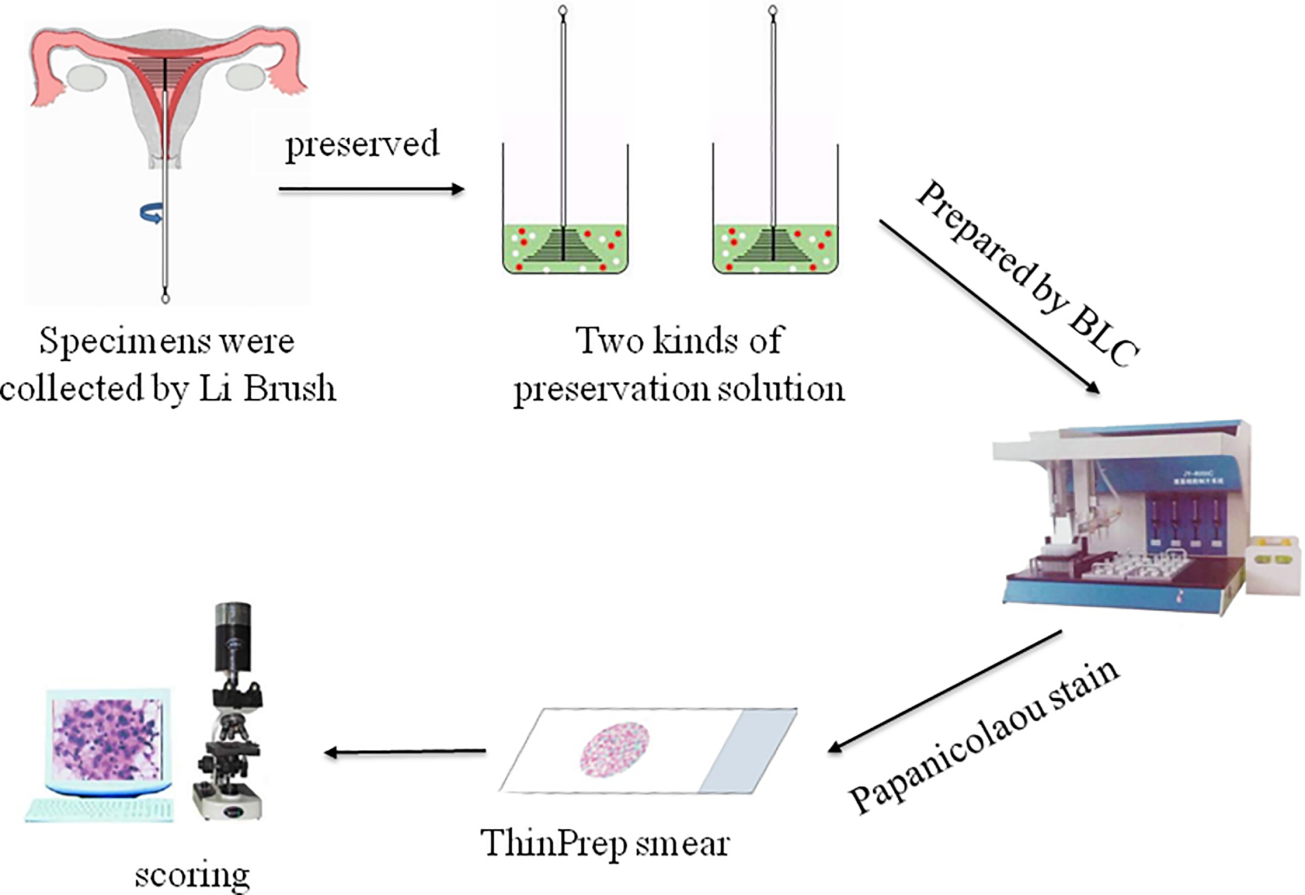
Flowchart of assessing the novel endometrial-cell preservation solution for endometrial brush sampler. LBC: liquid-based cytology.

This study protocol was approved by the Ethics Committee of the First Affiliated Hospital of Xi'an Jiaotong University (Approval number: XJTU1AF2015LSL-007–1), and written informed consent was obtained from all patients participated in the study. The study comprised a total of 10 specimens and endometrium was collected from 5 patients (30–50 years old). The flowchart of this method was shown in Fig 1.

### Configuration of cell preservation solution

The novel endometrial-cell preservation solution was mixed uniformly with the following reagents, 3.4 g ammonium chloride, 4.0 g sodium citrate, double distilled water and dissolving ammonium chloride and sodium citrate, 2 ml formaldehyde, shaking and adding double distilled water and making up total volume to 100 ml.

The patented control preservation solution was mixed with the following reagents, 4 g sodium citrate and 0.85 g sodium chloride, pure water and dissolving sodium citrate and sodium chloride completely, 2 ml formaldehyde and 1 ml glacial acetic acid, 50 ml 95% ethanol, shaking and adding pure water and making up total volume to 100 ml, and mixing uniformly.

### The capacity of dissolving red blood cells

Anti-coagulated peripheral blood samples were collected from the Clinical Laboratory in the First Affiliated Hospital of Xi'an Jiao Tong University, and preserved in different cell preservation solutions. We used 5 centrifuge tubes with different preservation solutions, labeled as A, B, C, D and E. A and C were filled with the novel endometrial-cell preservation solution, B and D were added acetic acid glacial adjusting pH to 4.5 on the basis of the novel endometrial-cell preservation solution, E was filled with the patented liquid-based cell preservation solution. A and B had been kept for 2 months, C, D and E were prepared immediately. All centrifuge tubes kept the same blood cells and preserved at room temperature for 1 day.

### Collection of cytological specimens

The endometrial cytological samples were obtained from women before hysterectomy due to endometrial diseases of the First Affiliated Hospital of Xi'an Jiaotong University (09/2016 to 12/2016). All cytology specimens were collected with the Li Brush samplers (3) two times and rinsed into two kinds of cell preservation solutions separately. Briefly, the sheathed Li Brush was inserted to the level of uterine fundus. Its overlying sheath was retracted to expose the brush bristles to the uterine cavity. The brush was rotated clockwise 360°, about 5 or 10 circles. The sheath was replaced over the brush bristles in order to entrap the collected cells. The closed assembly was removed from the uterus. The sheath was retracted to expose the brush into 10mL cell preservation solution immediately. The sheath was held firmly and the brush was moved in and out of the sheath with a bottle-brushing motion to clean it of adherent cells and tissue [5, 13].

### Preparation and staining

The vials were then sent to the Pathology Department of the First Affiliated Hospital of Xi'an Jiaotong University, processed for cytology assessment using the ZP-C automated slide preparation system. The vials were inserted into the automated slide processor, which prepared an endometrial smear in a thin layer in 90 seconds which within a microscopic field measuring 2 cm in greatest dimension. One slide was prepared for each case. The slides were stained with the Papanicolaou stain[14].

### Scoring system

A good procedure should meet the following conditions. General observation, there was no granular precipitation at the bottom of sample, and the supernatant was pale red. On low-power examination, endometrial cells distributed uniformly on LBC preparations, the background typically was clean. On high-power examination, cells morphology was integrity, cells inconspicuous nucleoli were present and diagnostic cells should exist [15, 16]. The above characteristics were analyzed by semi-quantitative scoring system (Table 1)[17]. The two experienced pathological physicians read blindly the cases.

**Table 1:** Scorning system.

Cytological features	Scores			
	0	1	2	3
**Cellularity**	Zero	Scanty	Adequate	Abundant
**Background** **blood debris**	Zero	Occasional	Good amount	Abundant
**Informative background**	Absent	Present	—	—
**Monolayer**	Absent	Occasional	Good amount	—
**Cell architecture**	Non-recognized	Moderately recognized	Well recognized	—
**Nuclear details**	Poor	Fair	Good	Excellent
**Cytoplasmic details**	Poor	Fair	Good	Excellent

### The capacity to preserve endometrial cells

The endometrial cytological samples were kept at room temperature for 2 days in the novel preservative solution and the patent solution separately. Then, after preparing and dyeing, the effect was evaluated under a microscope by scoring system.

### The preserved period of solutions

The endometrial cytological samples were preserved in the novel preservative solution and the patent solution separately and kept at room temperature for 2 days and 7 days. Then, after preparing and dyeing, the effect of two time points was evaluated under a microscope following the scoring system.

### The stability of solutions

The novel endometrial-cell preservation solution was kept at room temperature for 1 month and preserved endometrial cytological samples. After preparing and dyeing, the effect was evaluated under a microscope following the scoring system compared with the new prepared solution.

### Diagnostic coincidence rates

After the collection of endometrial cells by Li Brush in the novel preservative solution, the tissue of endometrium was performed routine histopathologic evaluation as control.

### Statistical analysis

Statistical analysis was performed using the Wilcoxon signed rank test on the SPSS program (SPSS 18.0). P < 0.05 was set as difference [17].

## Results

### Lysis of red blood cells

**Fig 2:**
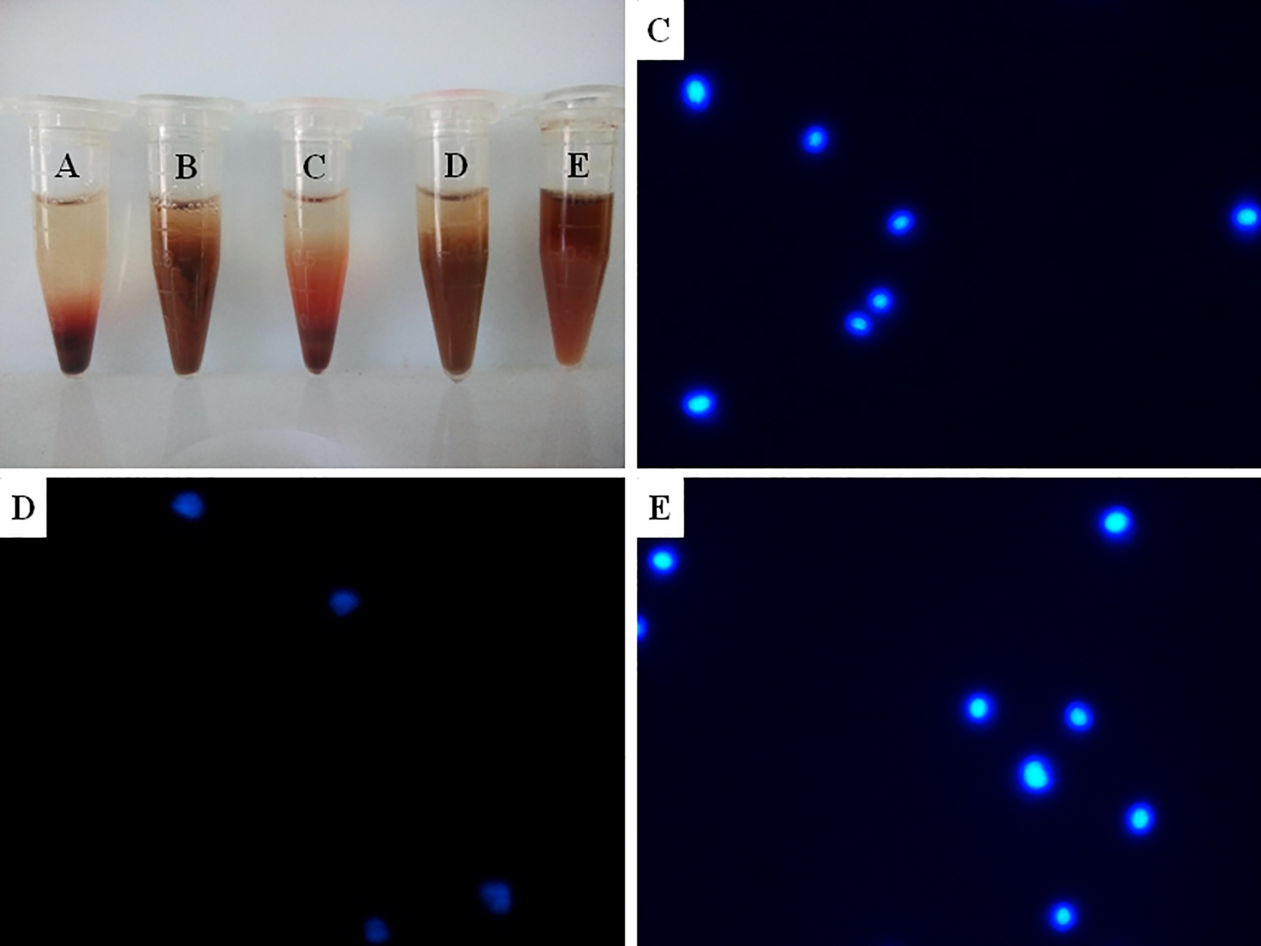
**Centrifuge tube A, B, C, D and E contained three kinds of cell preservation solutions which had two kinds of preservation period.** Tube A and C were novel endometrial-cell preservation solution, tube B and D added glacial acetic acid solution adjusting pH on the basis of A and C respectively, tube E was the patented liquid-based cell preservation solution. Tube A and B had prepared for 2 months, tube C, D and E for 0 day. Picture C, D and E were results of Centrifuge tube C, D and E for DAPI fluorescent staining respectively. ×400.

Blood samples were used to examine the effect of dissolving red blood cells and fixing nuclears of three kinds of cell preservation solutions. The preservation solutions of centrifuge tubes A and C split red cells effectively. No granular precipitation was at the bottom of the tubes and the supernatant was pale red. Rare red blood cells were on the conventional smear. The morphology of DAPI fluorescent staining cells was clearly visible. The ability A solution lysis of red blood cell was poorer than that of C solution. B and D solution had floc, DAPI fluorescence staining showed different patterns of cell debris, were not available; Centrifuge tube E contains a number of granular precipitation at the bottom of the tube, there were more red cell fragments on the smear, the morphology of DAPI fluorescent staining cells was clearly visible (Fig 2). The novel preservation solution can split red blood cells and fixed nuclear cells.

### Preservation of endometrial cells

**Fig 3:**
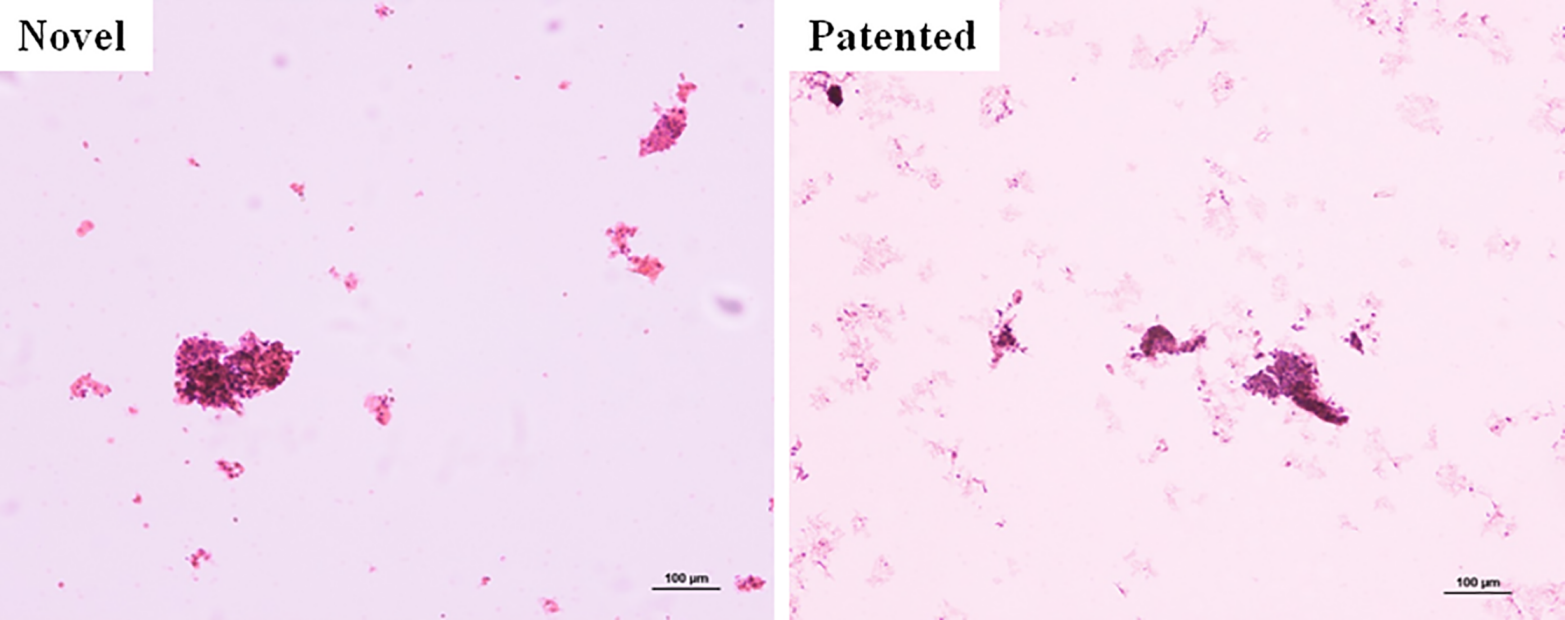
Endometrial-cells on low-power examination from two kinds of cell preservation solutions. Novel, novel endometrial-cell preservation solution. Patented, patented liquid-based cell preservation solution. ×100.

In the 10 specimens, there were no inadequate collections and all LBC preparations were available. On LBC preparations, endometrial cells from the two kinds solutions were all visible clearly (Fig 3), but samples from the novel endometrial cells preservation solution had more cell quantity, less red blood cell fragments, and the background was cleaner compared with control group. It also showed that the patented liquid-based cell preservation solution cannot lysis red blood cells completely, and cells were admixed with debris and blood. The red blood cells could be removed in the novel endometrial-cell preservation solution, at the same time endometrial-cells morphology were integrity. Although the novel endometrial-cell preservation solution showed cellularity and absence of blood and debris expressed by no statistically significant differences (p = 0.063 and 0.102 respectively) (Table 2, S1 Table).

**Table 2:** Wilcoxon signed rank test statistics novel endometrial-cells preservation solution vs. patented liquid-based cell preservation solution.

Statistics(n = 10)	Cellularity	Blood	Background	Monolayers	Architecture	Cytoplasm	Nucleus
**Z**	-1.857^b^	-1.633^a^	0	0	0	0	0
**Asymptotic, signed test** **(2 tailed)**	0.063	0.102	1	1	1	1	1

(a) Based on negative ranks;

(b) Based on positive ranks; p<0.05 is statistically significant.

### Preserved period of solutions

**Fig 4:**
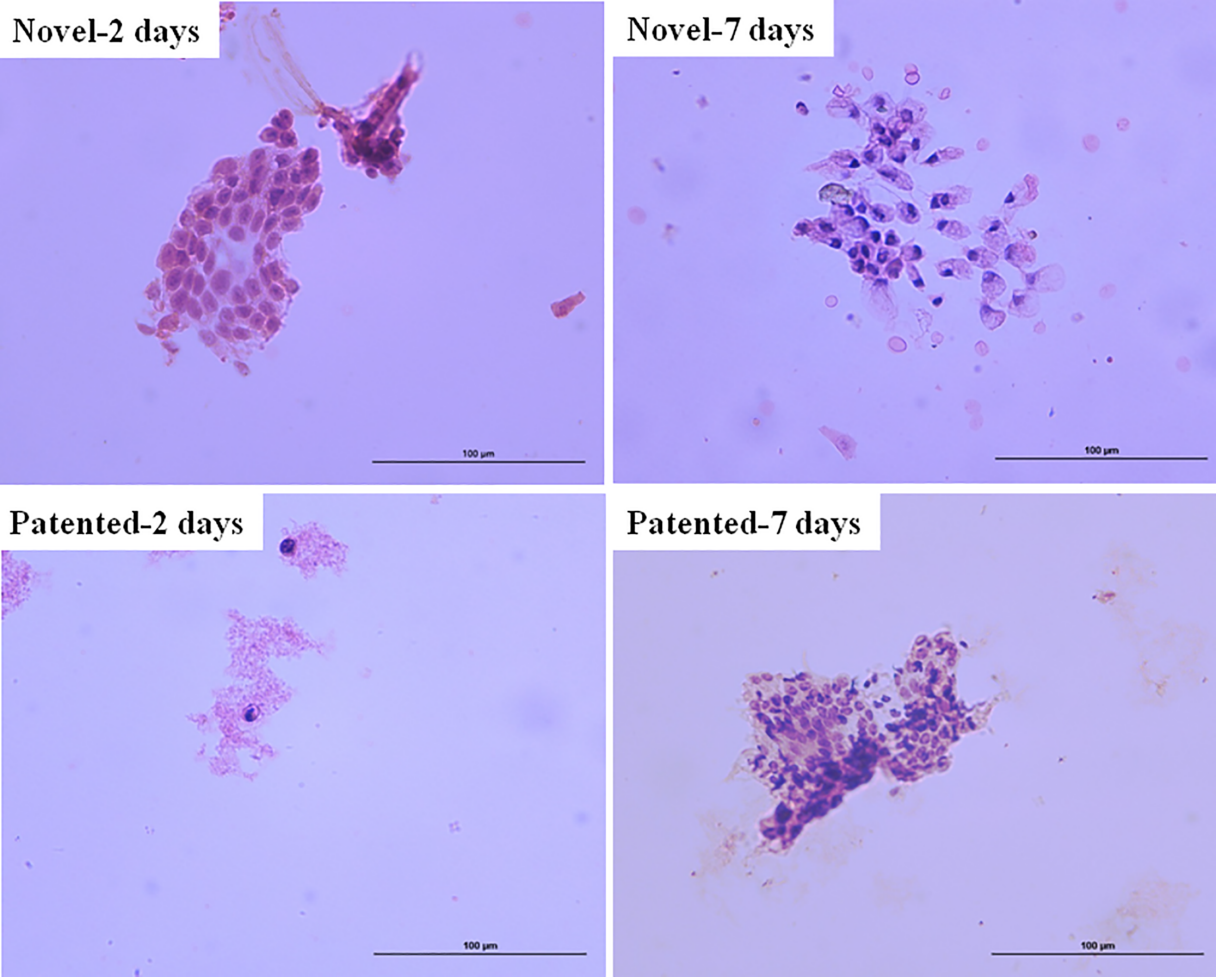
Endometrial cells on high-power examination from two kinds of cell preservation solutions. The cells were preserved for 2 days and 7 days respectively. ×400.

Under the microscope, the two time points all had a considerable effect on the fixed endometrial cells, which can maintain the integrity of the endometrial cell morphology, cells inconspicuous nucleoli were present, and had diagnostic cells, besides samples from the patented liquid-based cell preservation solution had more red blood cell debris (Fig 4). The results showed that the same preservation period of two kinds of liquids was similar.

### Stability properties of solution

The effect of preservation and fixed cells in solution placed for long time was equivalent to the new prepared. The results showed that the novel endometrial-cell preservation solution was stable.

### Diagnostic coincidence rates

The histologic diagnosis (used as the gold standard) of these hysterectomy specimen had two types separated (1) high-grade (atypical) hyperplasia, and (2) carcinoma. In the 10 LBC preparations, there were two types cytological diagnosis, which were consistent with histologic diagnosis. Results are presented in Table 3.

**Table 3:** Cyto-Histologic correlations for two kinds of preservation solution.

Histology	Cytology		
	Proliferative	High-grade-hyperplasia	Carcinoma
Proliferative	0	0	0
High-grade-hyperplasia	0	2	0
Carcinoma	0	0	3

## Discussion

Researchers fail to provide a reliable method for screening of the endometrial lesions partly because that the inappropriate sampling device and the techniques of cell fixation increase the difficulty in obtaining a result interpretation. In 1955, Clyman first reported the endometrial-cells collector named endometrial cannula used in endometrial cytology[17], due to sampling operation is difficult and often suffer from cervical and vaginal cell contamination, therefore, cannot be widely recognized by gynecological pathological physicians. In 1993, LC Tao developed Tao Brush endometrial cell collector which avoids the cell pollution outside uterine cavity and easy to operate, so recognized by the clinical gynecologists and widely used[5]. In some studies, the Tao brush has been reported to be a painless and alternative method for detecting endometrial lesions [18]. Endometrial-cell collector is simple, safe and painless, high quality and low price, which is gradually applied to the cytological screening of endometrial lesions. Li Brush, a self-designed endometrial brush collected endometrial samples, is used in this study. Li-brush is a new type of endometrial brush on the basis of the existing endometrial brush design, combined with the special shape of uterine cavity [3]. The biggest advantage is that you can collect all the cell samples of the uterine cavity, especially the corners and fundus of the uterus.

In 1996, LBC was approved by the American Food and Drug Administration (FDA) and widely used in the screening of cervical cancer, with known and established advantages over conventional cytology in nearly all cellular samples[19]. In 1997, LBC was applied to endometrial cytology, and achieved a diagnostic sensitivity as accurate as conventional preparations, especially for its excellent cell preservation and clearance of background which decreased the amount of inadequate diagnoses[13]. ThinPrep cytology combined with endometrial sampling could be a useful tool for the outpatient diagnosis of endometrial lesions, and reduced the number of unnecessary curettages[15].

Effective and stable cytology test is the key link to make accurate diagnosis of endometrial disease. Different from the histopathological diagnosis, there is limited number of cells to be detected in cytological smear. If the quality of smear does not pass, it will cause a lot of interference to the diagnosis of pathological physician, and may make a false judgment. There are many factors that interfere with smear quality, and the residual red blood cells remains is one of the main factors.

The number of red blood cells in endometrial samples obtained by endometrial brush is numerous, which is easy to cause smearing thickly and cells overlapping, blocking line of sight and impacting the diagnosis. Traditional cervical cell preservation solution cannot remove red blood cells effectively, in order to eliminate this phenomenon, we added appropriate components of hemolytic agent on the basis of a patented cell preservation solution, both destructing red blood cells and maintaining the integrity of other nuclear cells. The hemolytic agent is ammonium chloride. The effect is obvious, nucleoli are present and background is clear. This method does not affect the subsequent production of samples, concentrate other cells to a certain extent and endometrial-cells to the most extent, so as to improve the positive detection rate of cancer cells. The hemolytic mechanism of hemolytic agent in this paper is that, ammonium chloride is a strong acid and weak base salt, dissociate ammonia ion in the water, and Rh protein of red blood cell membrane has the function of transporting ammonia, large amounts of ammonia ions enter red blood cells, osmotic pressure increased in the red blood cells, promote water channel opening, a large number of water molecules into the red blood cells, hemolysis happens[20]. The lymphocytes or other nuclear cells are not damaged by ammonium chloride has no damage to while disrupting red blood cells. In our study, The expression of cellularity and absence of blood cells and debris is not statistically significant differences in two group(p = 0.063 and 0.102 respectively). The reason may be that the sample size was small, statistical evidence was insufficient. In addition, there is no cervical mucus in endometrial samples obtained by Li Brush, so we removed the chemical composition which break down proteins in the liquid-base cell preservation solution to reduce the cost.

In previous studies, we used anticoagulant blood to verify the effect of the novel cell preservation solution on lysis of red blood cells and the fixation effect of nuclear cells. In order to show the integrity of the fixed cells, we applied for DAPI fluorescent nuclear staining to verify the integrity of nuclei, the experimental results showed that two kinds of cell preservation solution can fix nucleated cells effectively. We tried to use the ice acetic acid to regulate the preservation solution pH, the same to the liquid-base cell preservation solution, which disrupted all cells.

## Conclusions

The novel endometrial-cell preservation solution is superior to the cervical liquid-base cell preservation solution with regard to clear background and cell preservation. If applied in clinic can improve the diagnosis accuracy of endometrial disease, reduce the cost of cell preservation solution. It is worth popularizing and applying.

## Supporting information

S1 Fig Lysis of red blood cells from different preservation solutions. A and C were filled with the novel endometrial-cell preservation solution, B and D were added acetic acid glacial adjusting pH to 4.5 on the basis of the novel endometrial-cell preservation solution, E was filled with the patented liquid-based cell preservation solution.
(JPG)

S2 Fig Lysis of red blood cells from different preservation solutions. S1 Fig marking methods in experiments.
(JPG)

S3 Fig The morphology of DAPI fluorescent staining cells from the preservation solutions of centrifuge tubes C. ×400. (JPG)

S4 Fig The morphology of DAPI fluorescent staining cells from the preservation solutions of centrifuge tubes A. ×400. (JPG)

S5 Fig The morphology of DAPI fluorescent staining cells from the preservation solutions of centrifuge tubes B. ×400. (JPG)

S6 Fig The morphology of DAPI fluorescent staining cells from the preservation solutions of centrifuge tubes D. ×400. (JPG)

S7 Fig The morphology of DAPI fluorescent staining cells from the preservation solutions of centrifuge tubes E. ×400. (JPG)

S8 Fig Endometrial-cells on low-power examination from the novel endometrial cells preservation solution. ×100. (JPG)

S9 Fig Endometrial-cells on low-power examination from the novel endometrial cells preservation solution. ×200. (JPG)

S10 Fig Endometrial-cells on low-power examination from the novel endometrial cells preservation solution. ×100. (JPG)

S11 Fig Endometrial-cells on low-power examination from the novel endometrial cells preservation solution. ×200. (JPG)

S12 Fig Endometrial-cells on low-power examination from the novel endometrial cells preservation solution. ×200. (JPG)

S13 Fig Endometrial-cells on low-power examination from the patented liquid-based cell preservation solution. 200. (JPG)

S14 Fig Endometrial-cells on low-power examination from the patented liquid-based cell preservation solution. ×200. (JPG)

S15 Fig Endometrial-cells on low-power examination from the patented liquid-based cell preservation solution. ×100. (JPG)

S16 Fig Endometrial-cells on low-power examination from the patented liquid-based cell preservation solution. ×100. (JPG)

S17 Fig Endometrial-cells on low-power examination from the patented liquid-based cell preservation solution. ×200. (JPG)

S18 Fig Endometrial cells on high-power examination from the novel endometrial cells preservation solution. ×400. (JPG)

S19 Fig Endometrial cells on high-power examination from the novel endometrial cells preservation solution. ×400. (JPG)

S20 Fig Endometrial cells on high-power examination from the novel endometrial cells preservation solution. ×400. (JPG)

S21 Fig Endometrial cells on high-power examination from the novel endometrial cells preservation solution. ×400. (JPG)

S22 Fig Endometrial cells on high-power examination from the novel endometrial cells preservation solution. ×400. (JPG)

S23 Fig Endometrial cells on high-power examination from the patented liquid-based cell preservation solution. ×400. (JPG)

S24 Fig Endometrial cells on high-power examination from the patented liquid-based cell preservation solution. ×400. (JPG)

S25 Fig Endometrial cells on high-power examination from the patented liquid-based cell preservation solution. ×400. (JPG)

S26 Fig Endometrial cells on high-power examination from the patented liquid-based cell preservation solution. ×400. (JPG)

S27 Fig Endometrial cells on high-power examination from the patented liquid-based cell preservation solution. ×400. (JPG)

S1 Table Scoring results of the 10 specimens by semi-quantitative scoring system. (PDF)
