# Supplementary figures and images for: A novel solution configuration on liquid-based endometrial cytology
Source: PLoS One. 2018 Feb 5;13(2):e0190851. doi: 10.1371/journal.pone.0190851 (PMC5798778; doi:10.1371/journal.pone.0190851)

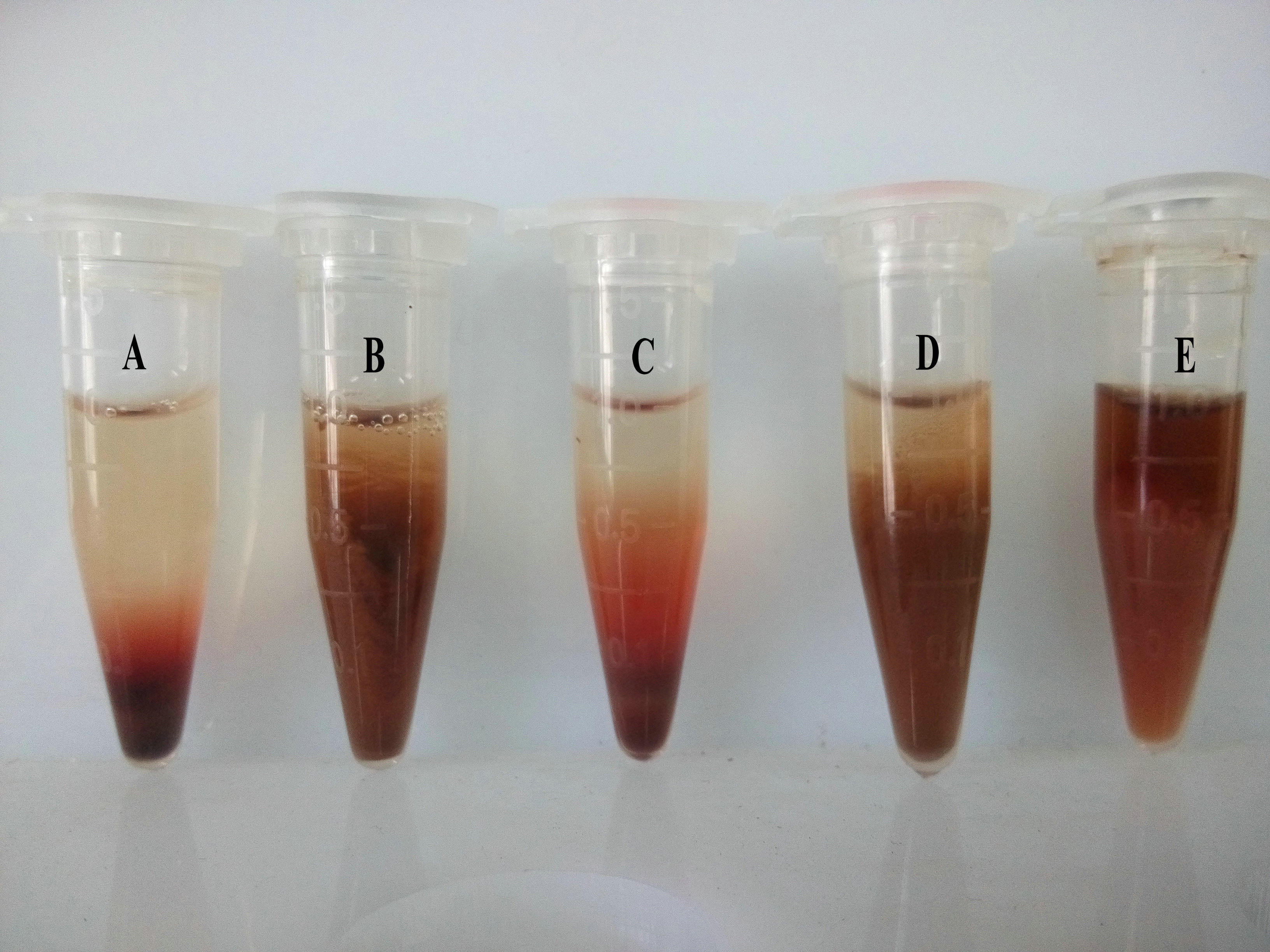

Supplement: S1 Fig — A and C were filled with the novel endometrial-cell preservation solution, B and D were added acetic acid glacial adjusting pH to 4.5 on the basis of the novel endometrial-cell preservation solution, E was filled with the patented liquid-based cell preservation solution. (JPG) [file pone.0190851.s001.jpg]

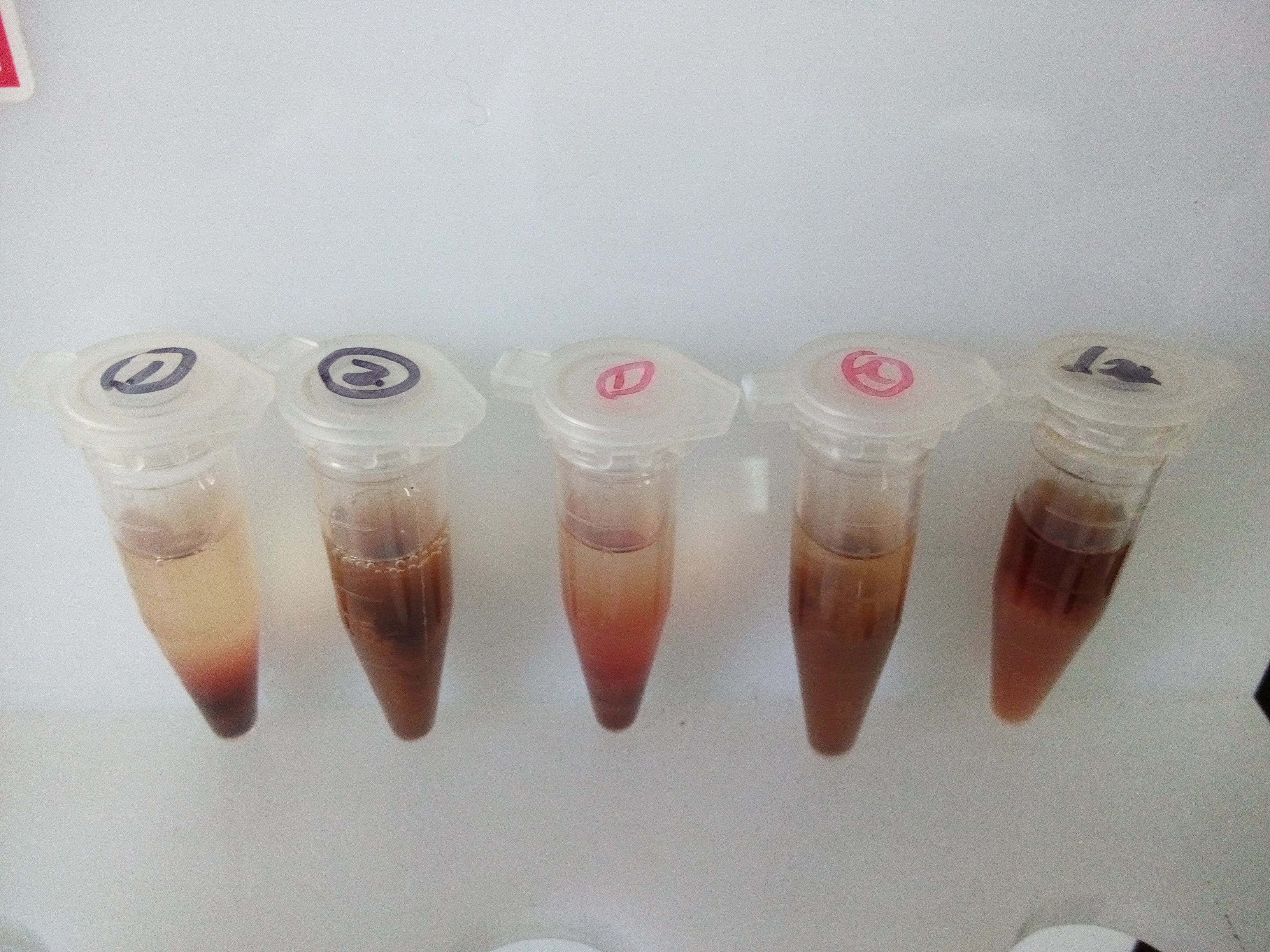

Supplement: S2 Fig — S1 Fig marking methods in experiments. (JPG) [file pone.0190851.s002.jpg]

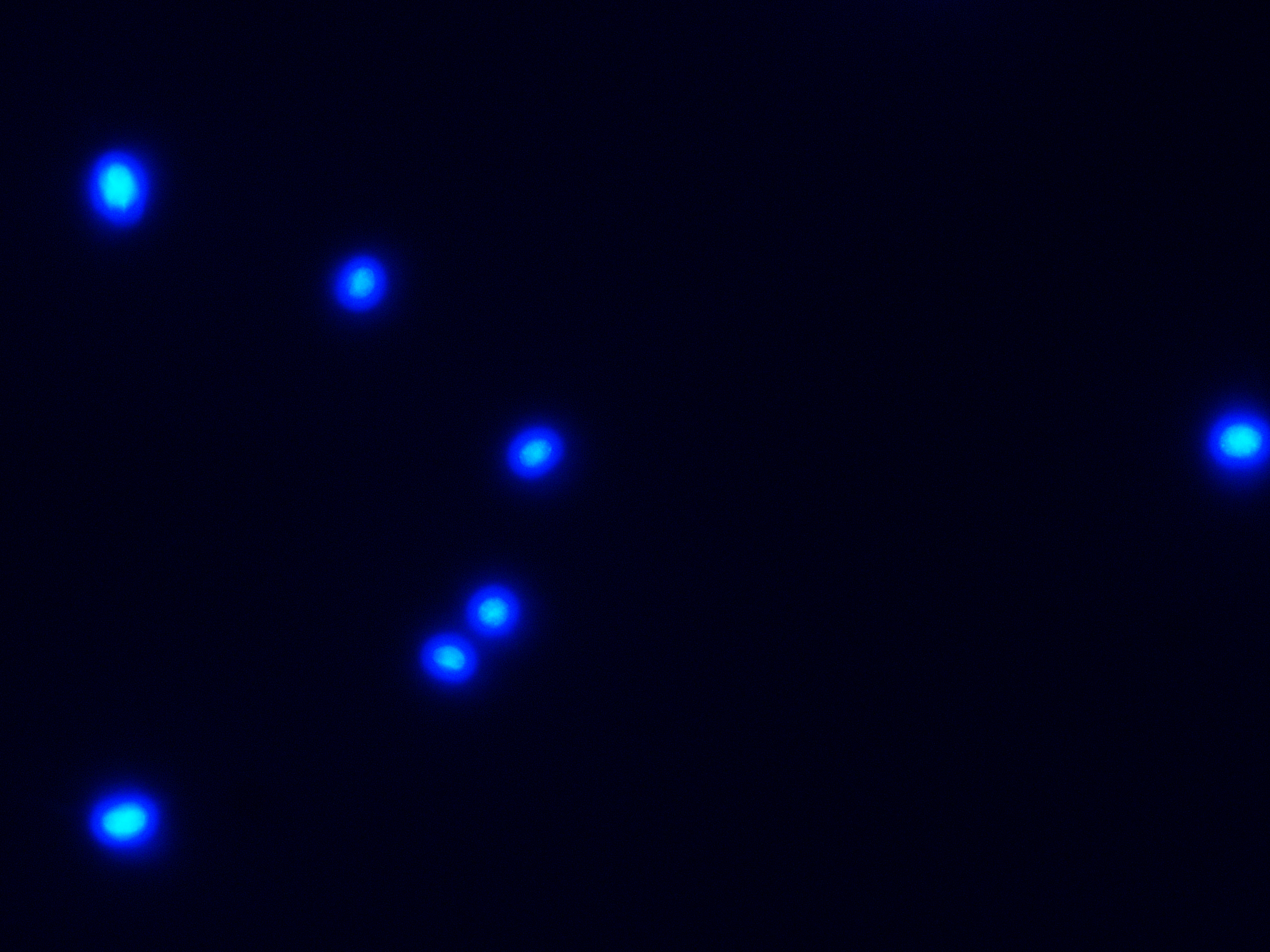

Supplement: S3 Fig — (JPG) [file pone.0190851.s003.jpg]

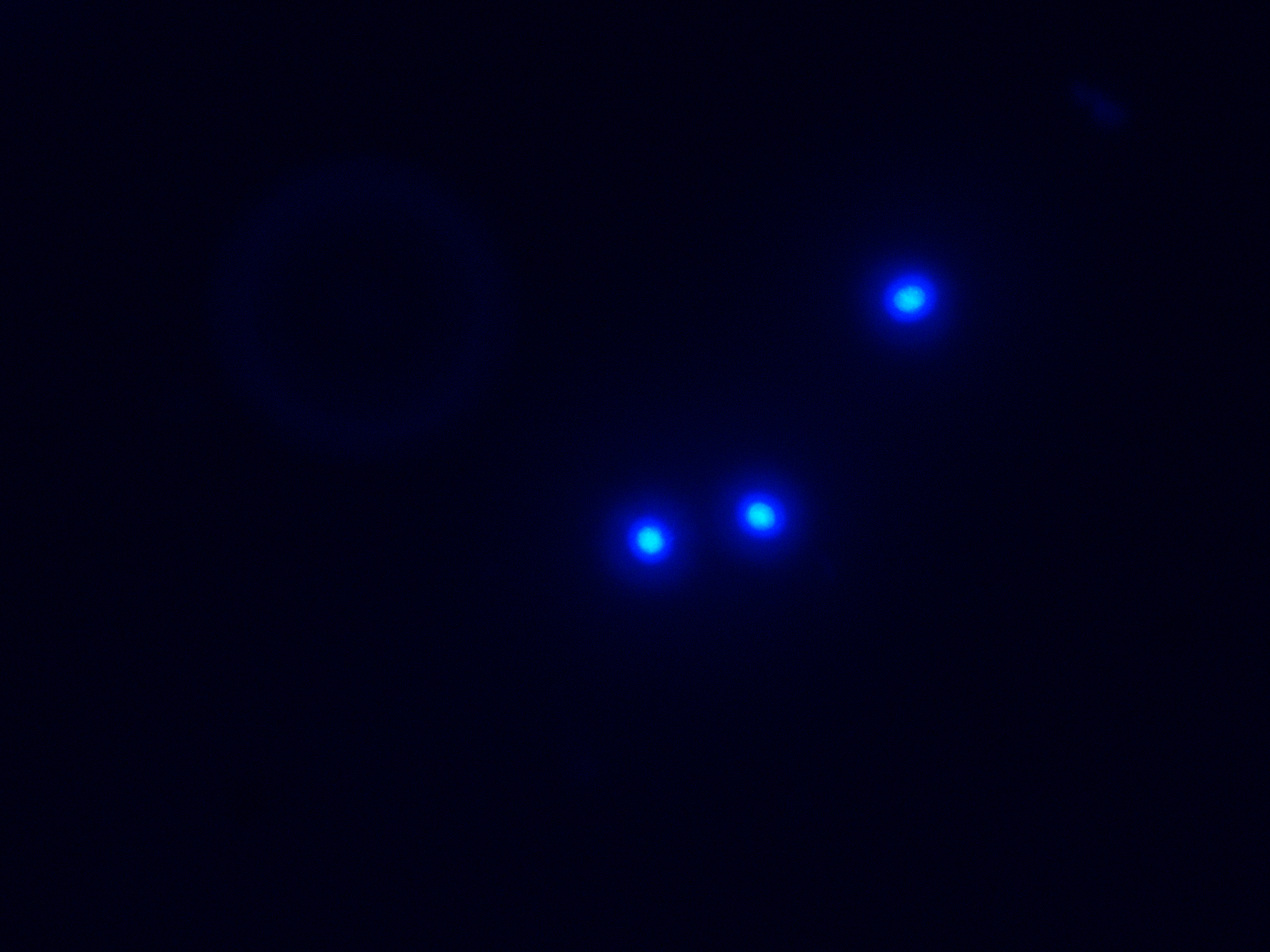

Supplement: S4 Fig — (JPG) [file pone.0190851.s004.jpg]

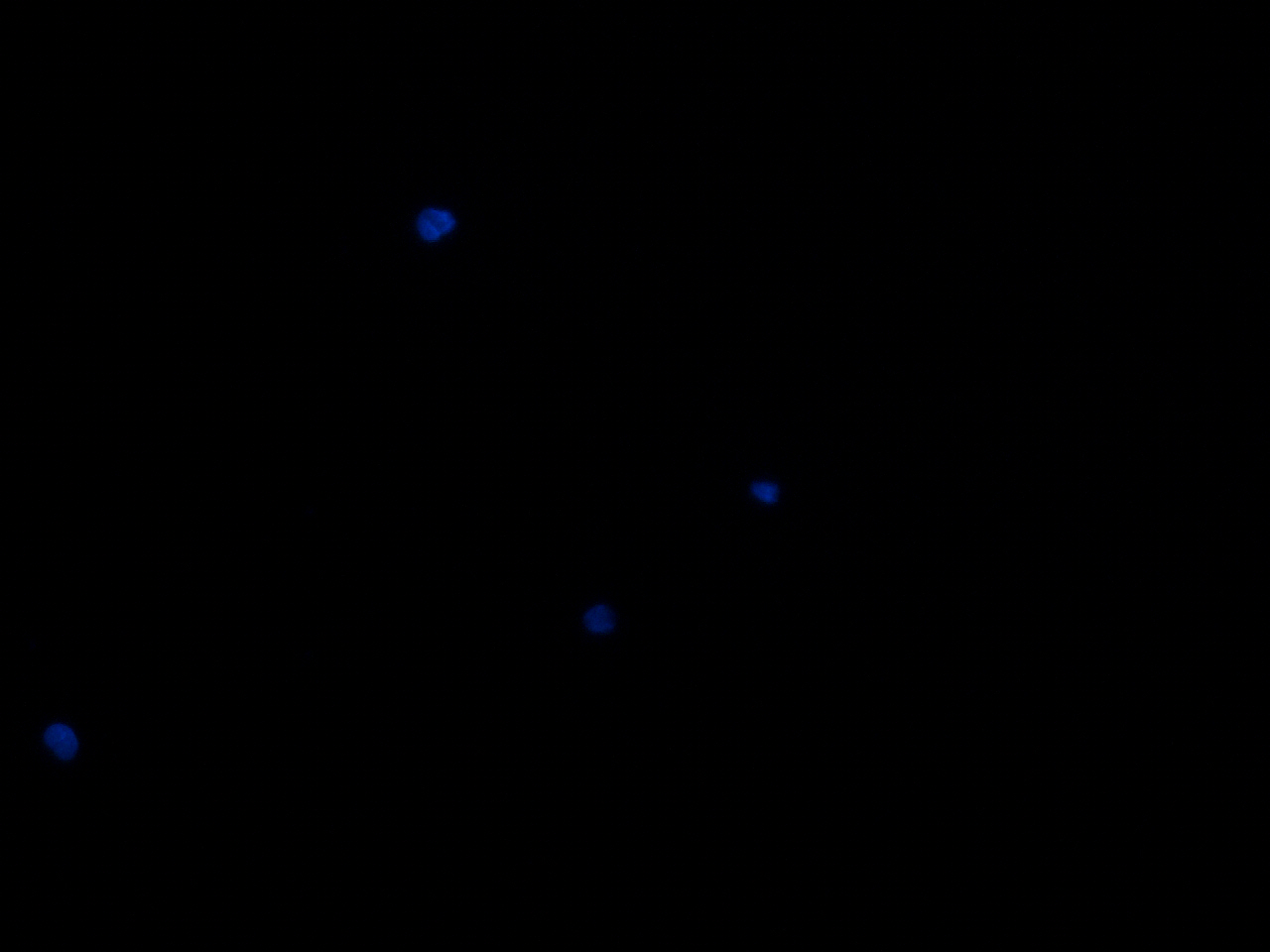

Supplement: S5 Fig — (JPG) [file pone.0190851.s005.jpg]

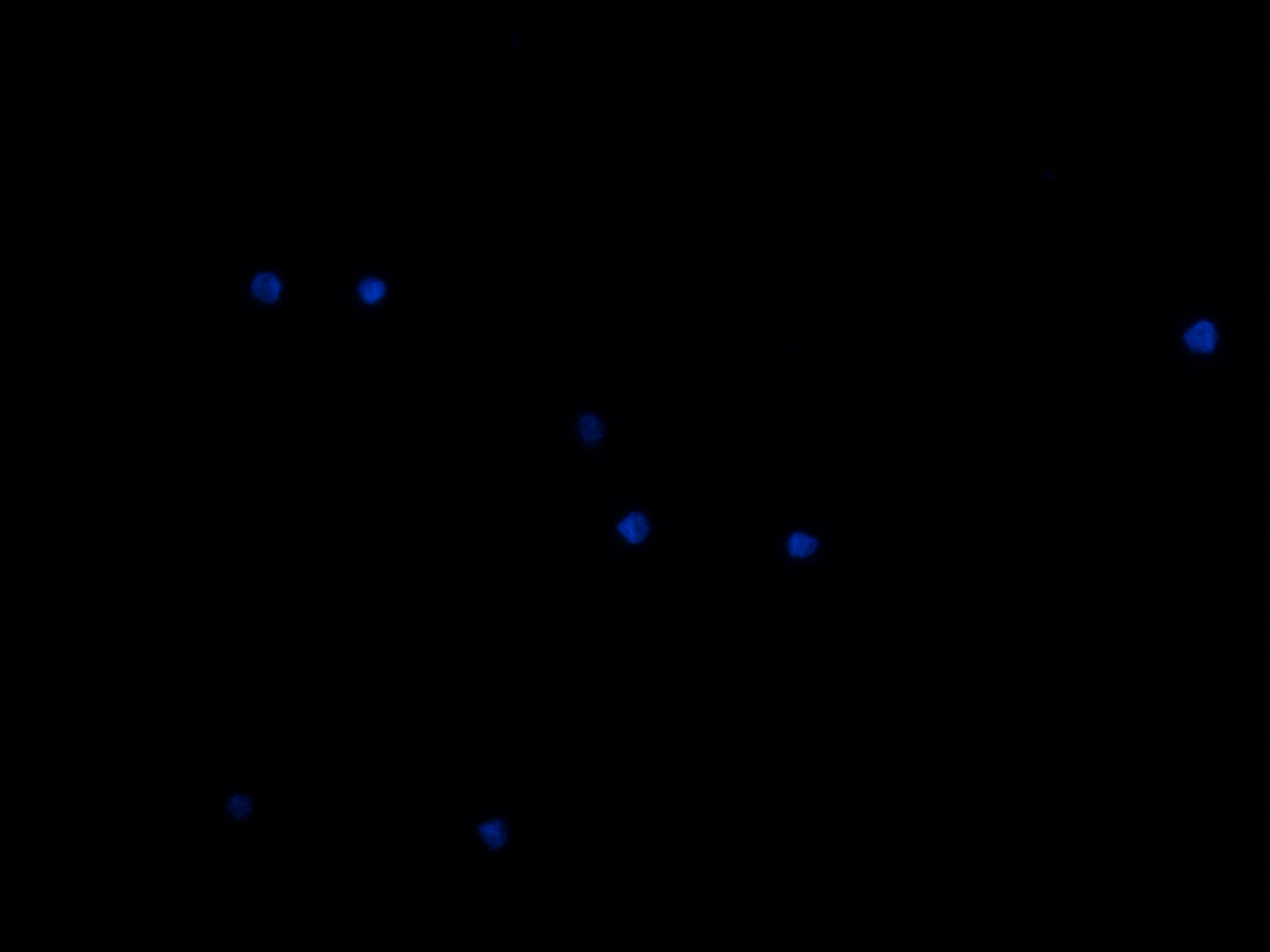

Supplement: S6 Fig — (JPG) [file pone.0190851.s006.jpg]

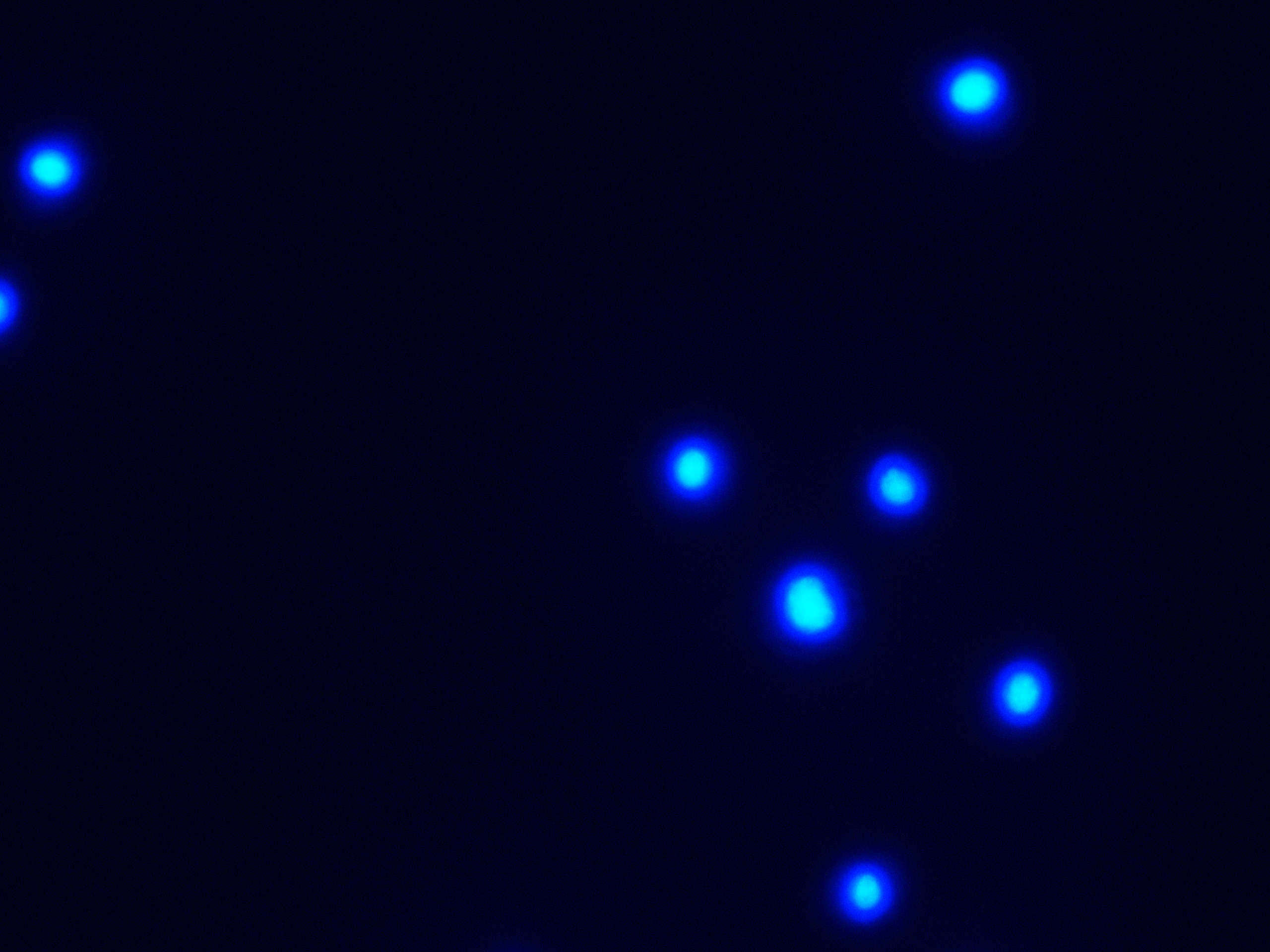

Supplement: S7 Fig — (JPG) [file pone.0190851.s007.jpg]

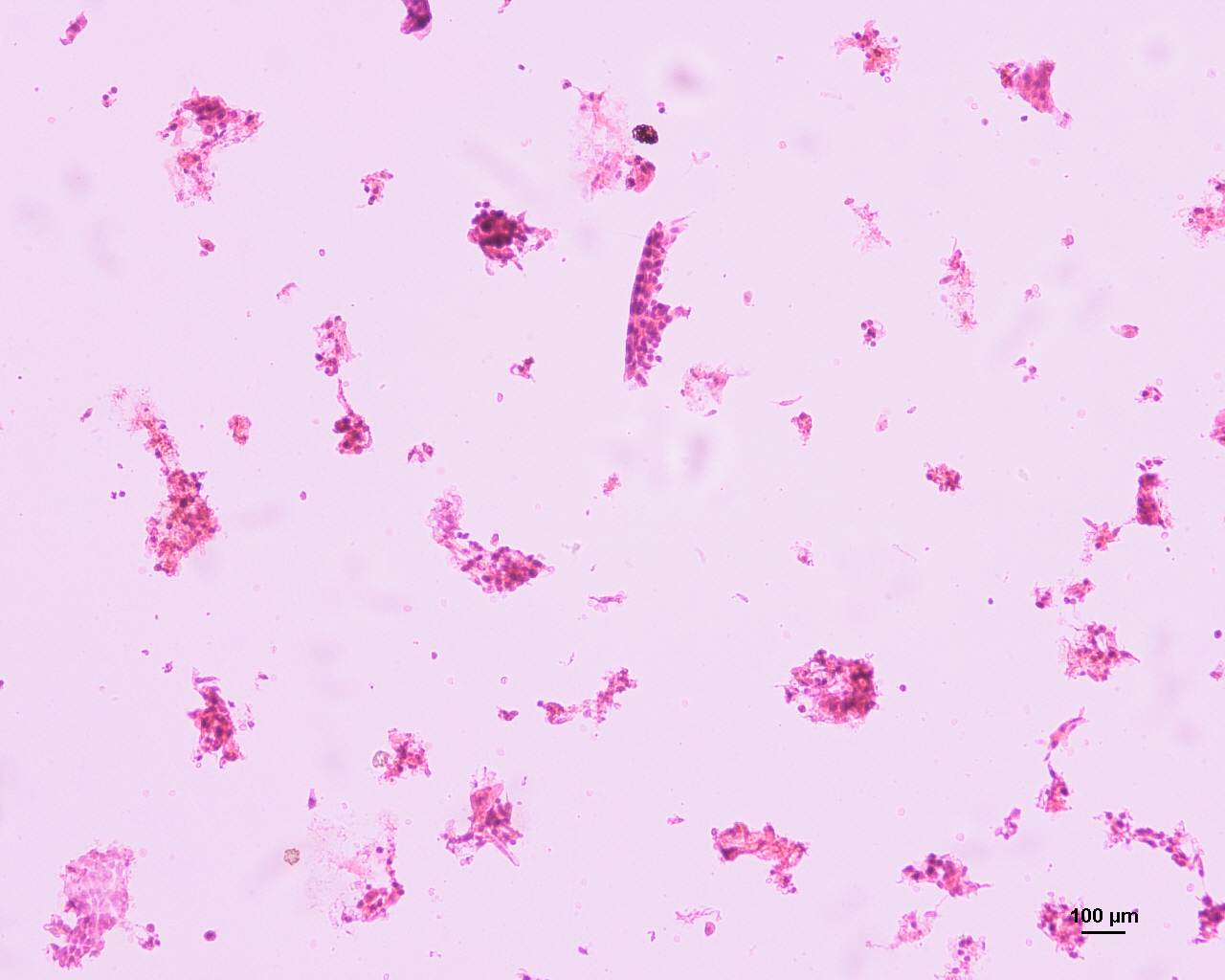

Supplement: S8 Fig — (JPG) [file pone.0190851.s008.jpg]

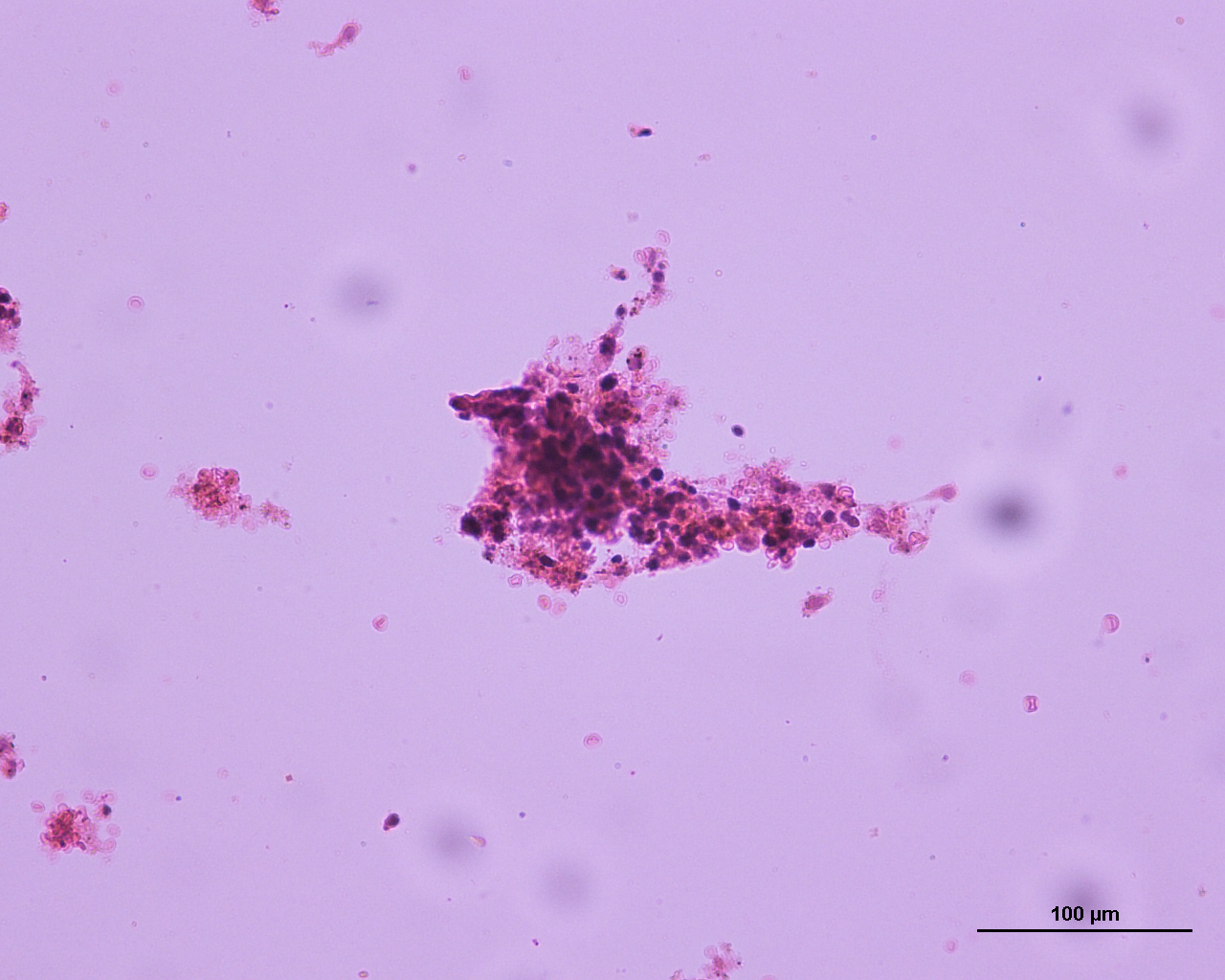

Supplement: S9 Fig — (JPG) [file pone.0190851.s009.jpg]

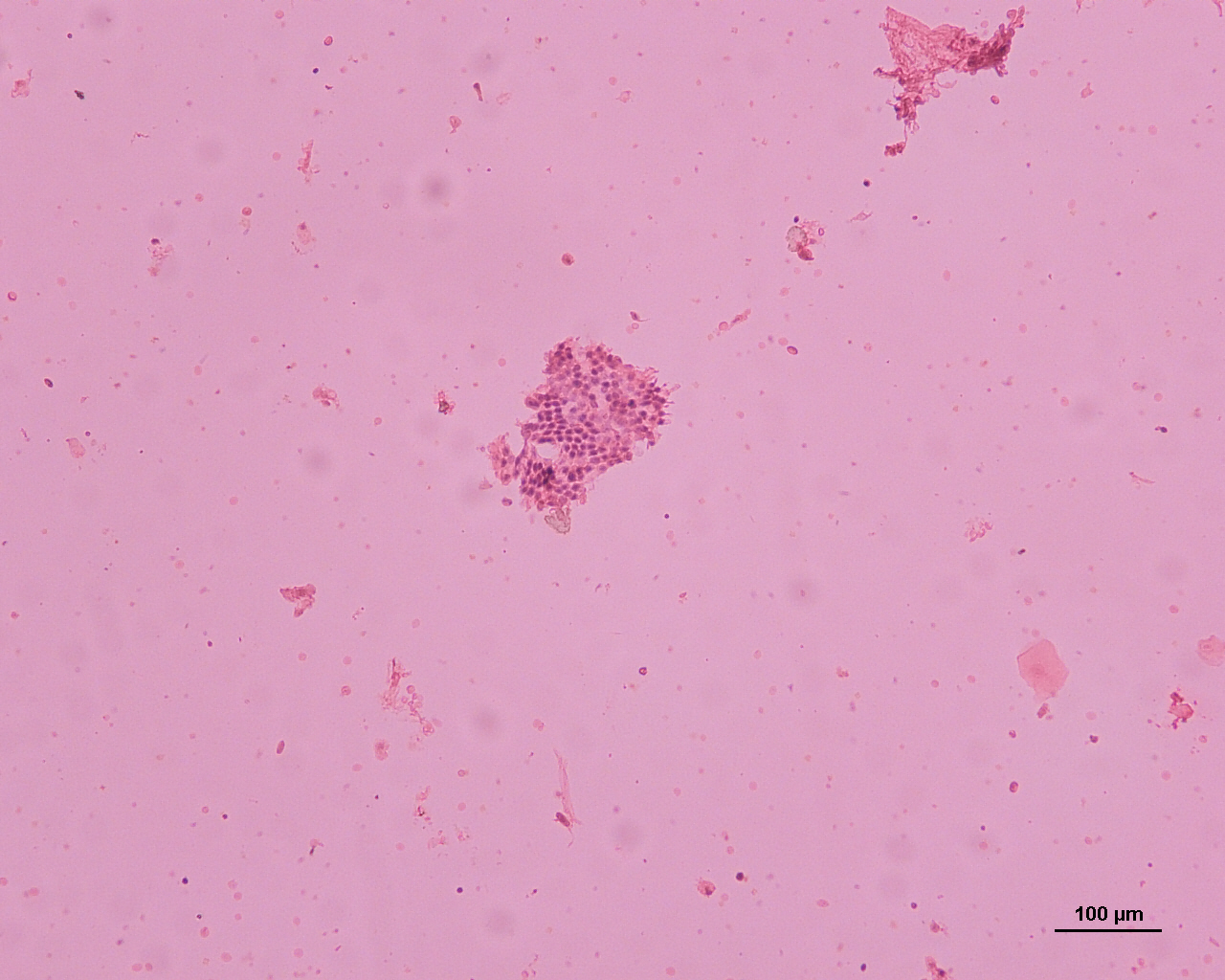

Supplement: S10 Fig — (JPG) [file pone.0190851.s010.jpg]

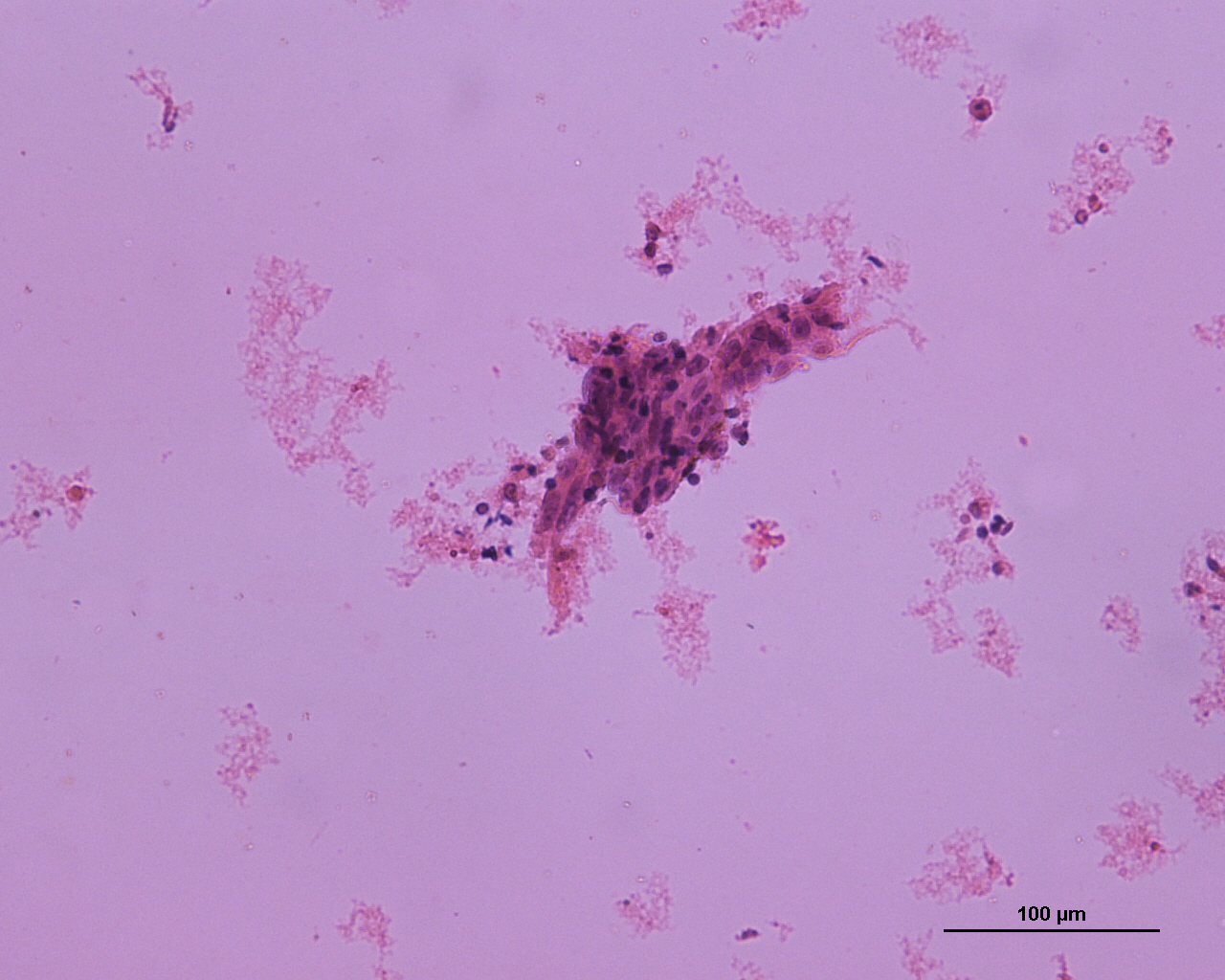

Supplement: S11 Fig — (JPG) [file pone.0190851.s011.jpg]

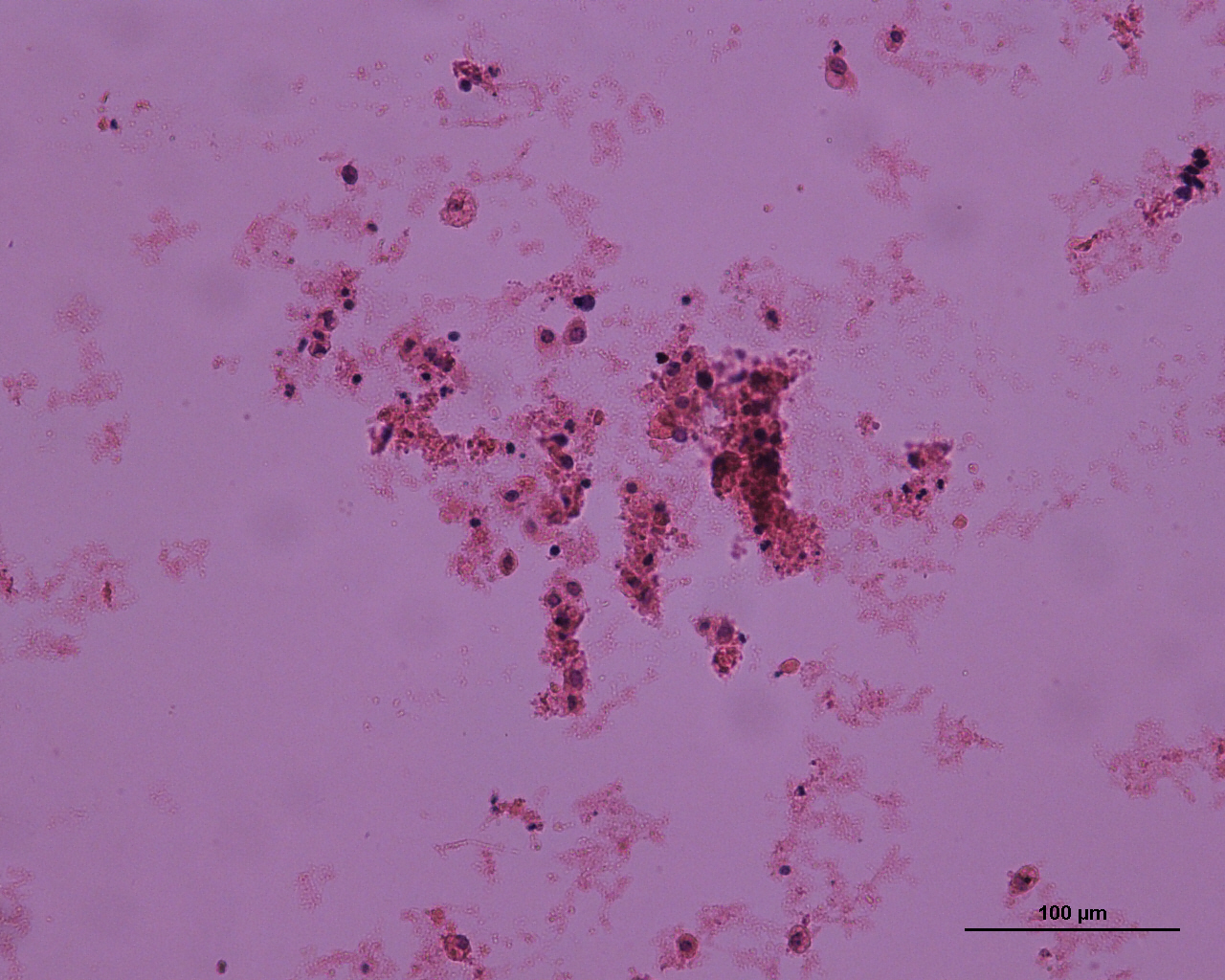

Supplement: S12 Fig — (JPG) [file pone.0190851.s012.jpg]

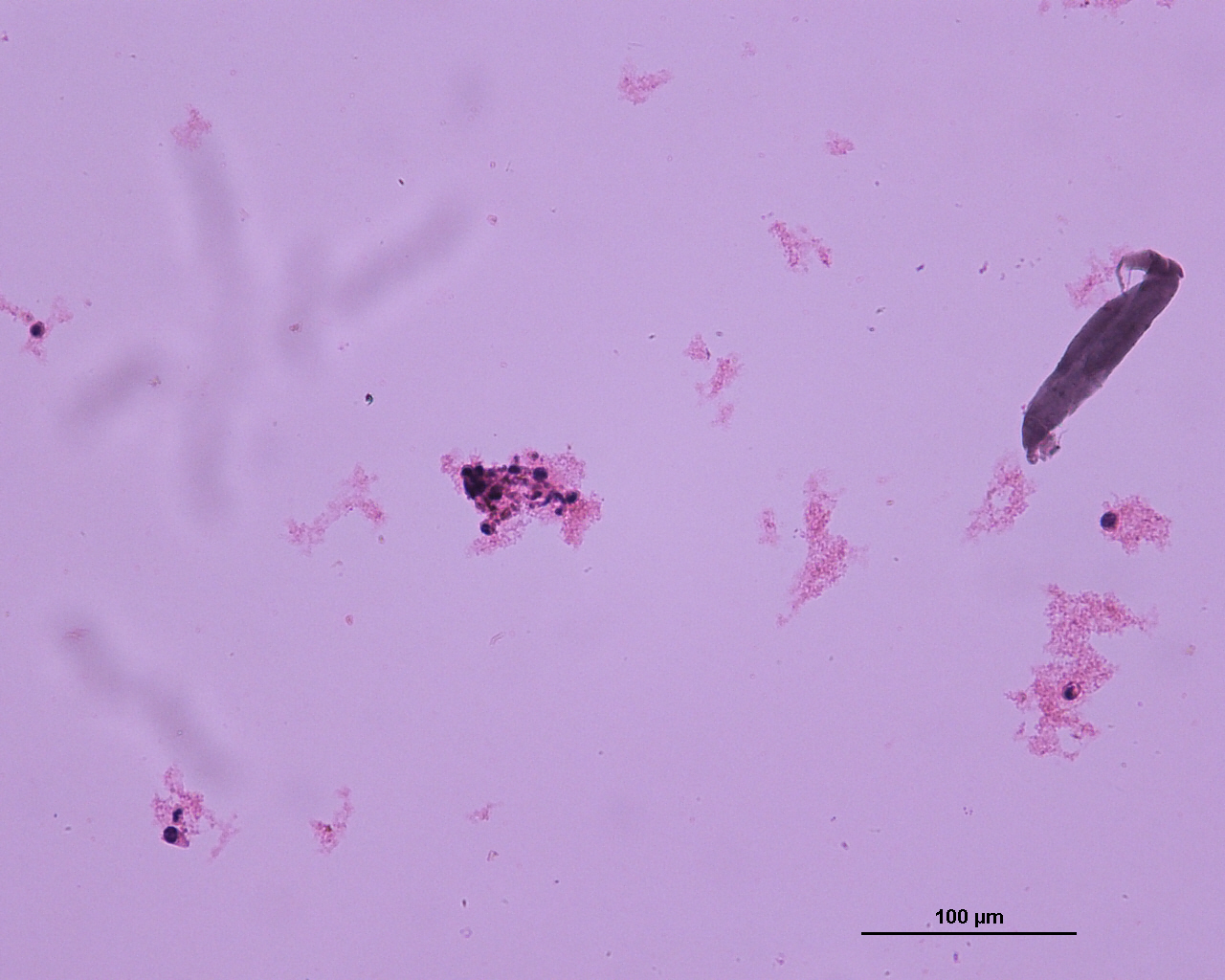

Supplement: S13 Fig — (JPG) [file pone.0190851.s013.jpg]

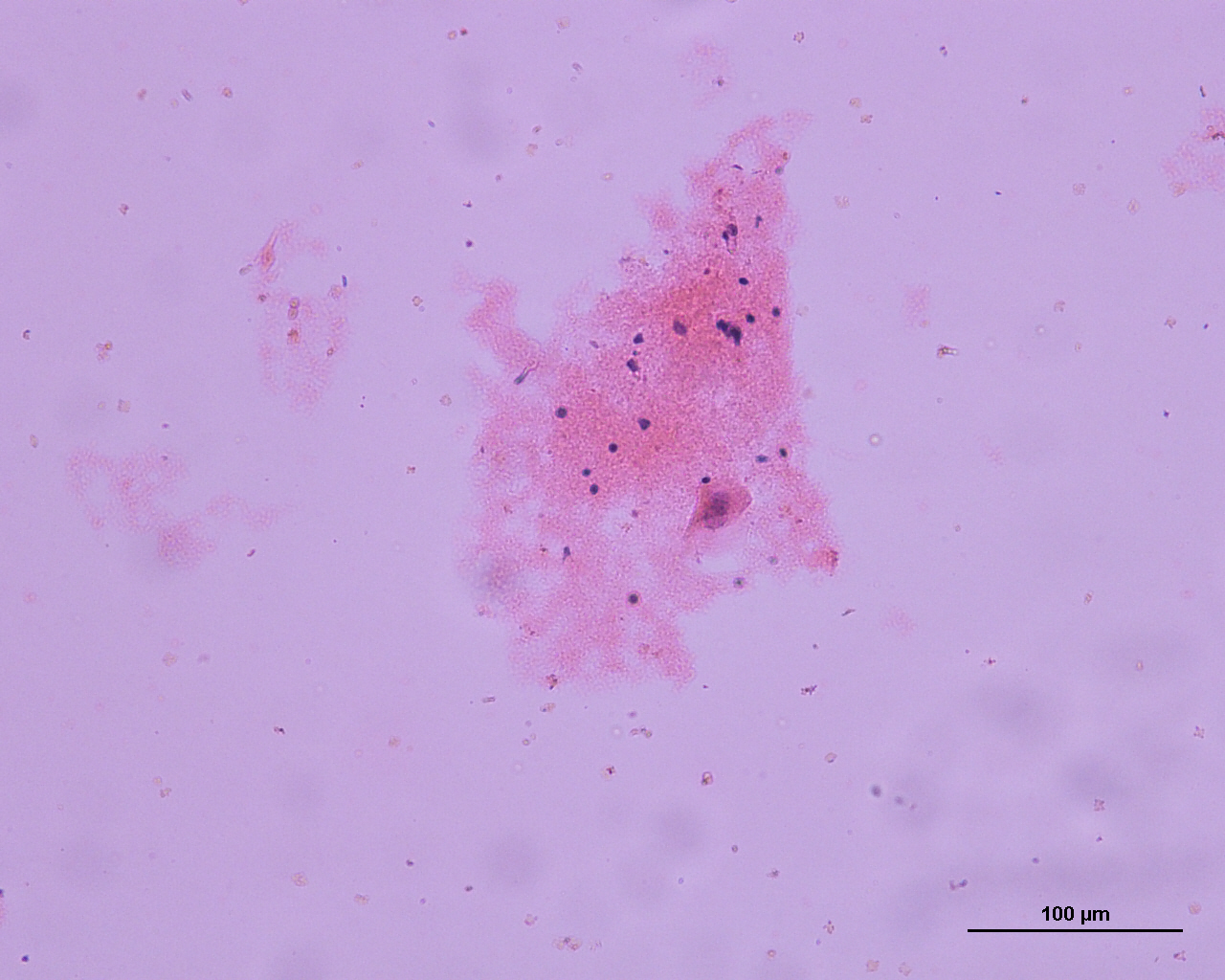

Supplement: S14 Fig — (JPG) [file pone.0190851.s014.jpg]

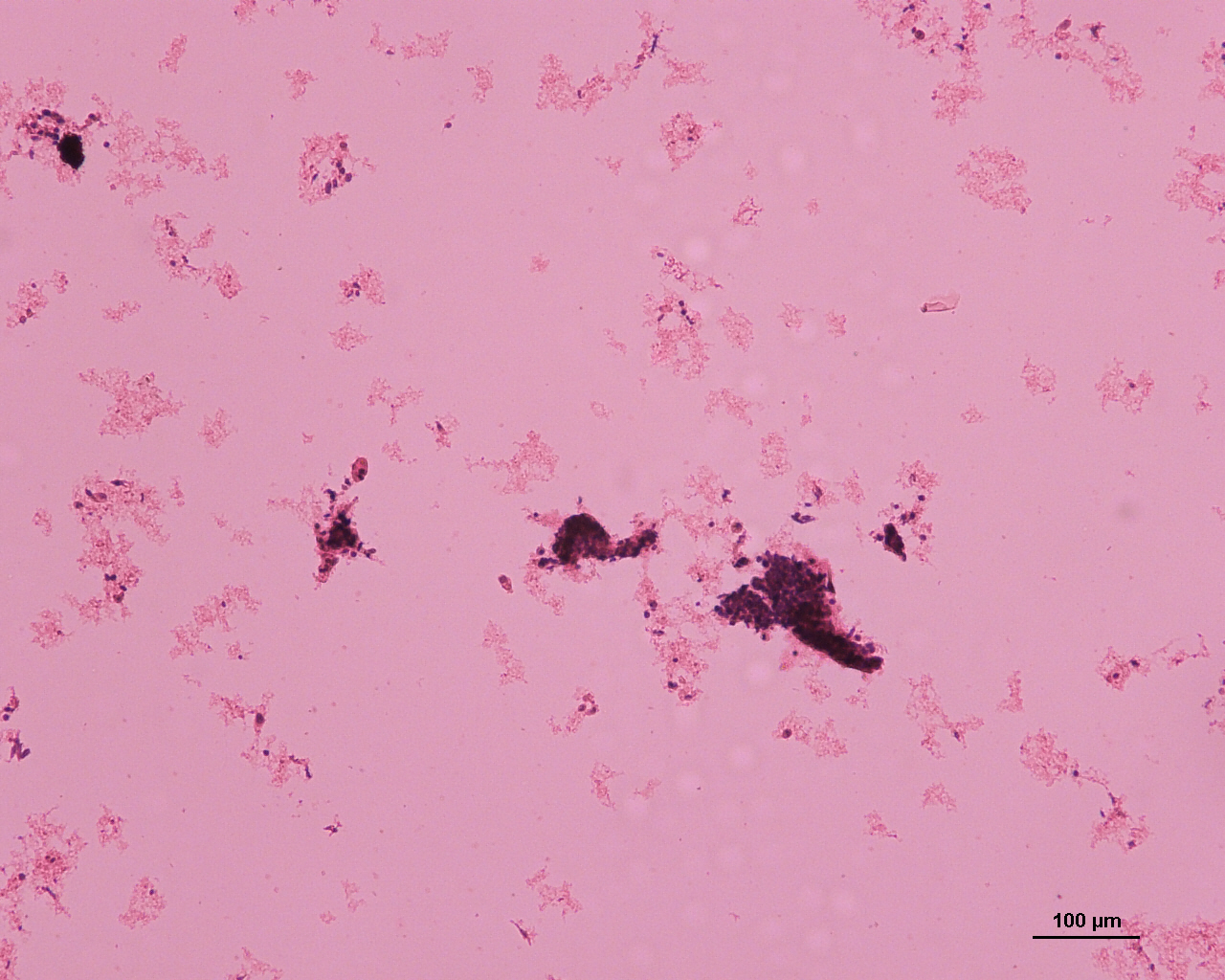

Supplement: S15 Fig — (JPG) [file pone.0190851.s015.jpg]

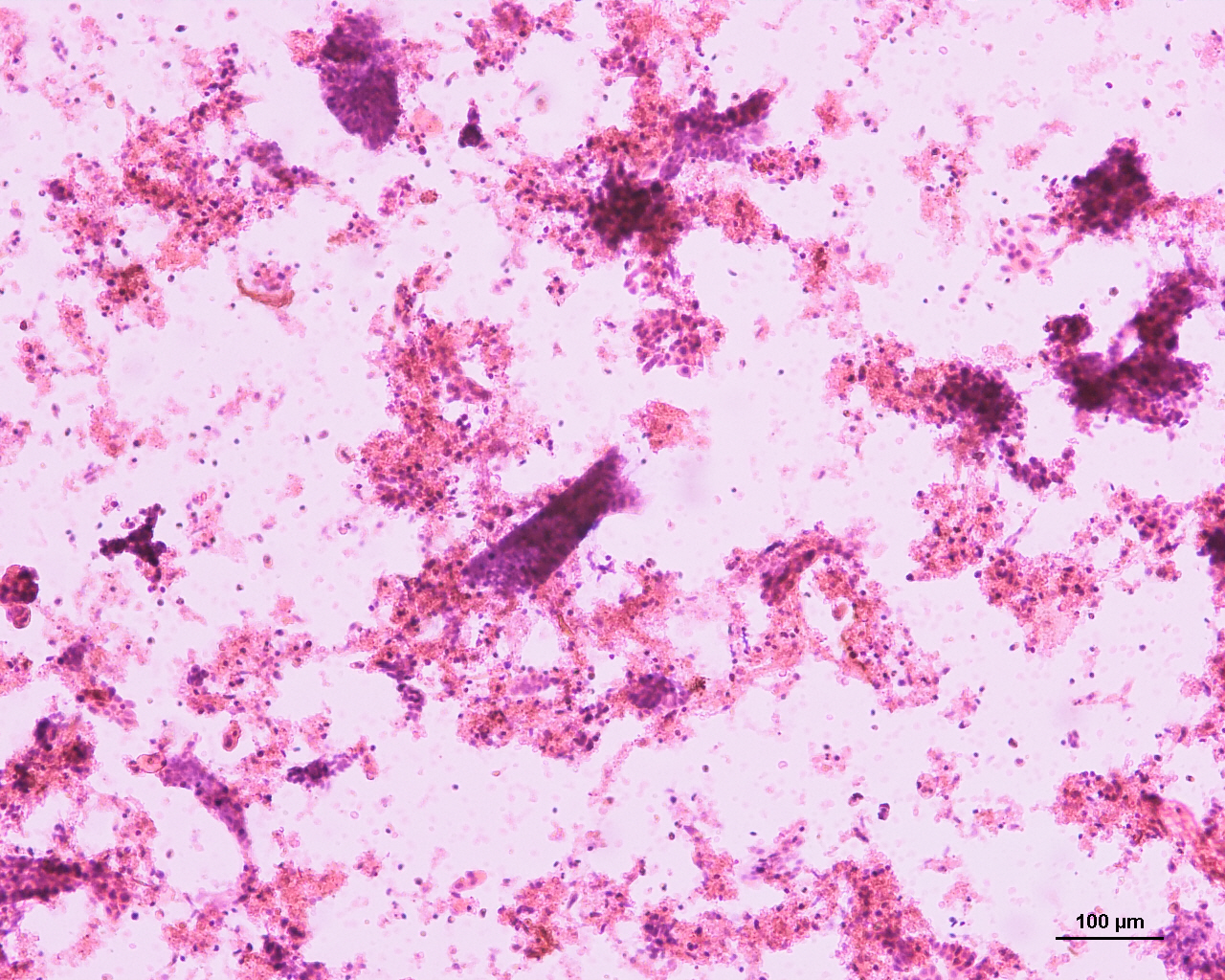

Supplement: S16 Fig — (JPG) [file pone.0190851.s016.jpg]

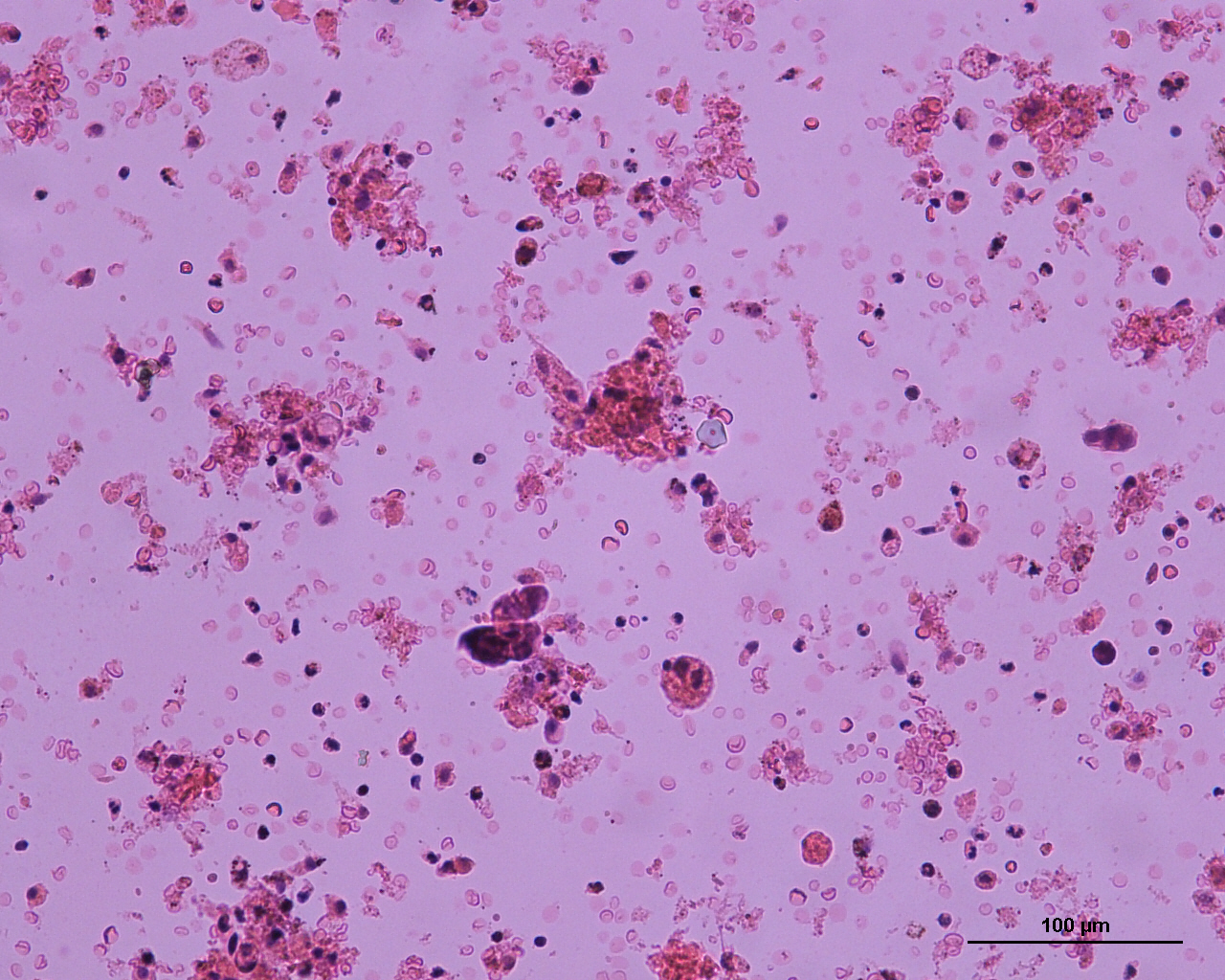

Supplement: S17 Fig — (JPG) [file pone.0190851.s017.jpg]

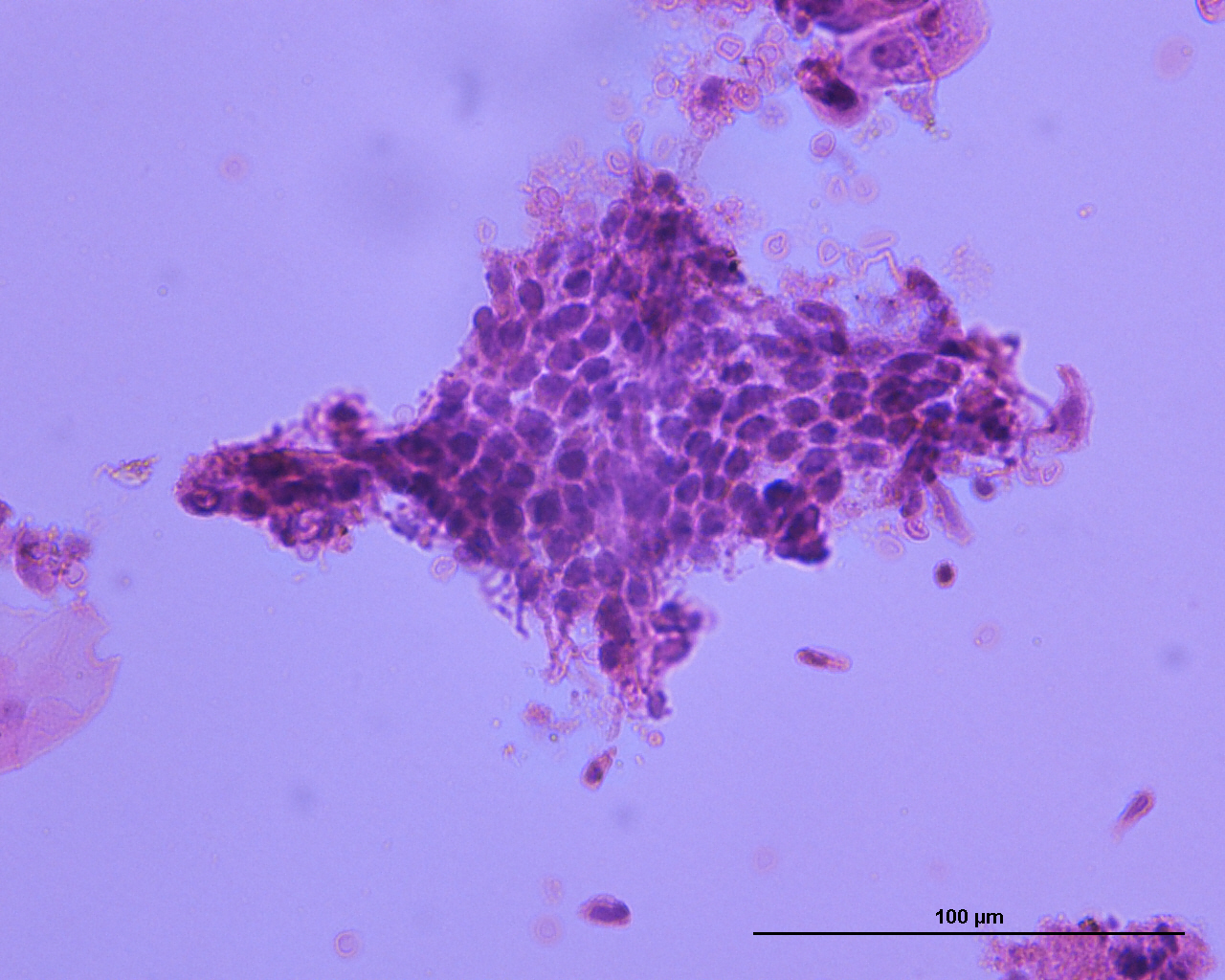

Supplement: S18 Fig — (JPG) [file pone.0190851.s018.jpg]

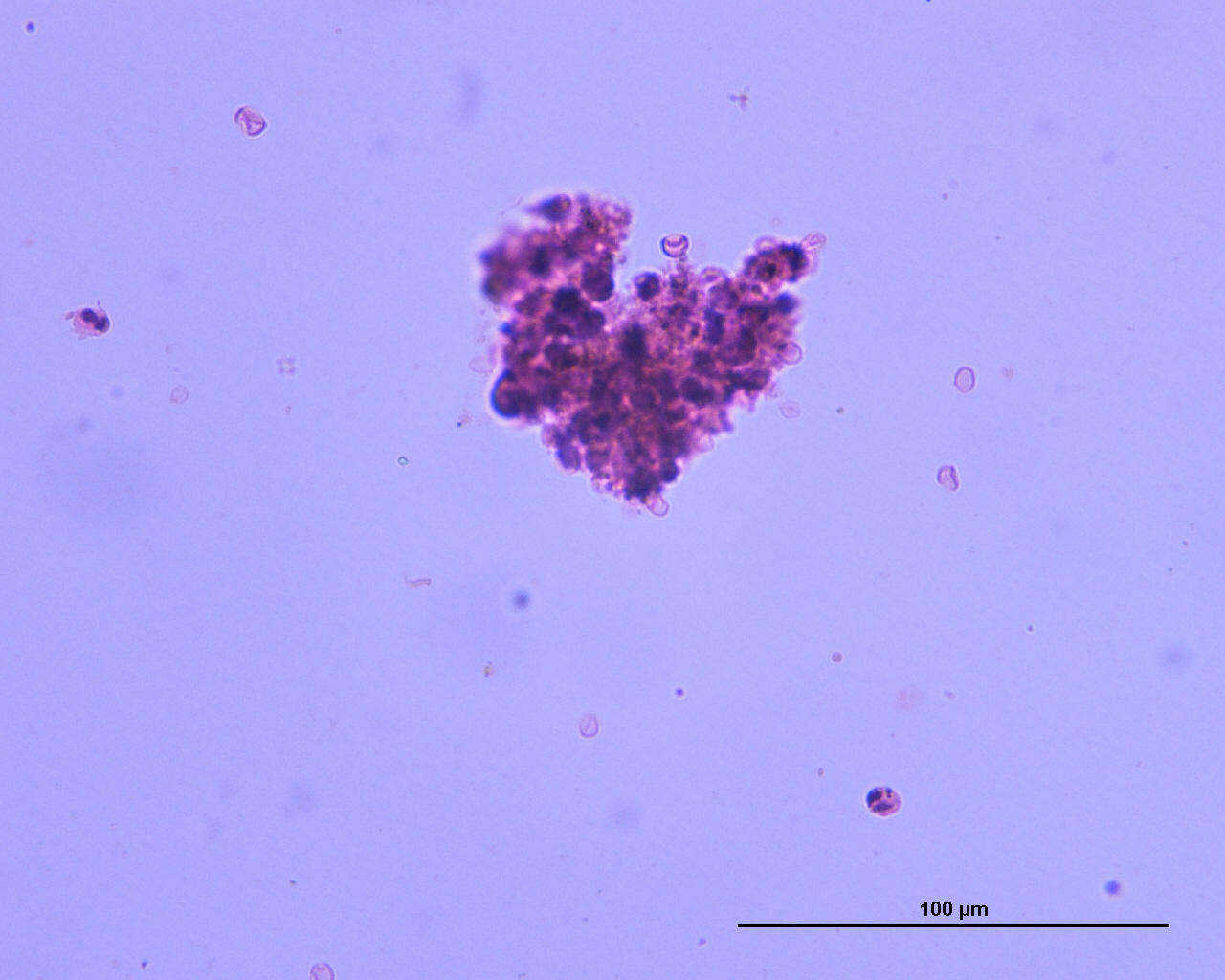

Supplement: S19 Fig — (JPG) [file pone.0190851.s019.jpg]

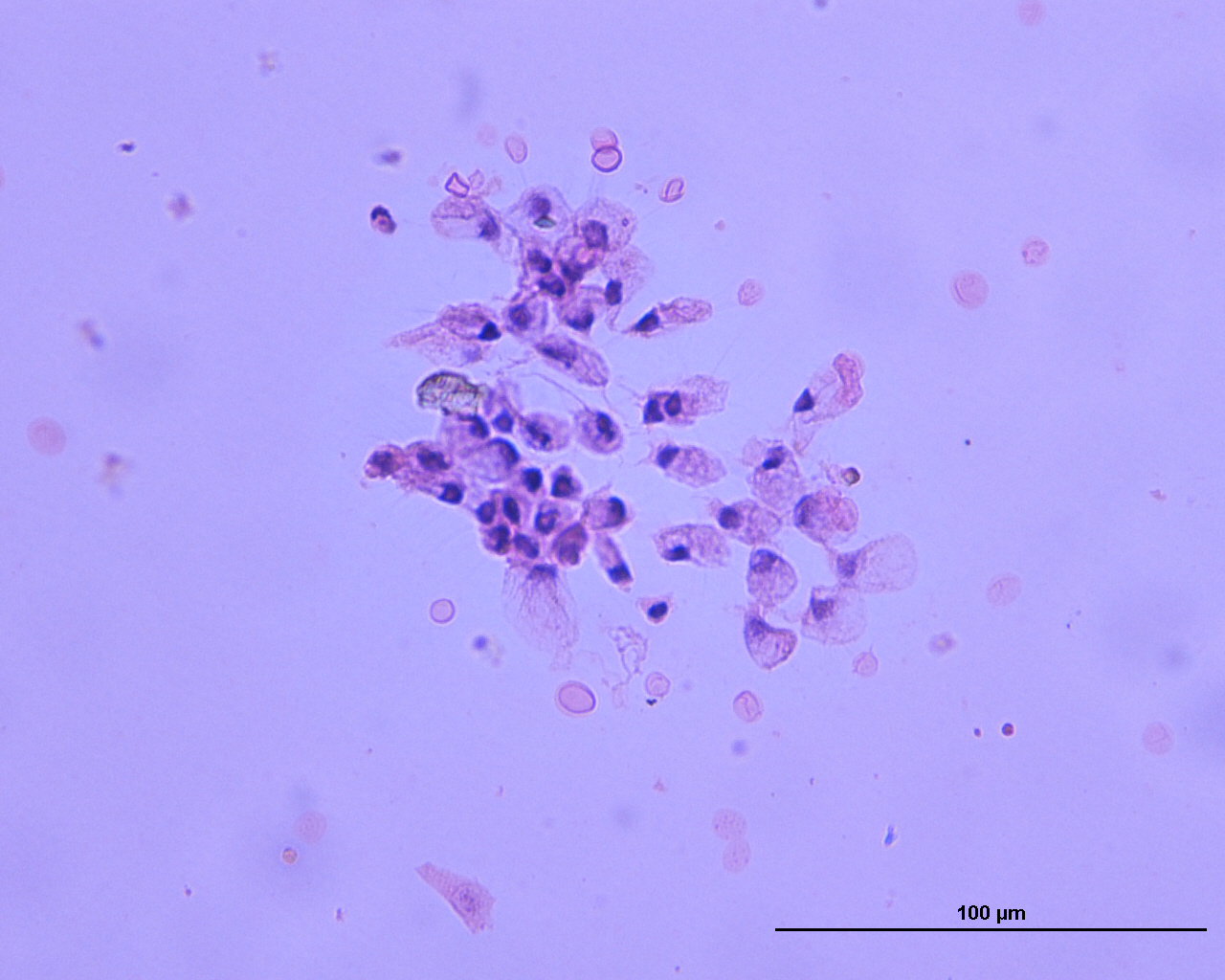

Supplement: S20 Fig — (JPG) [file pone.0190851.s020.jpg]

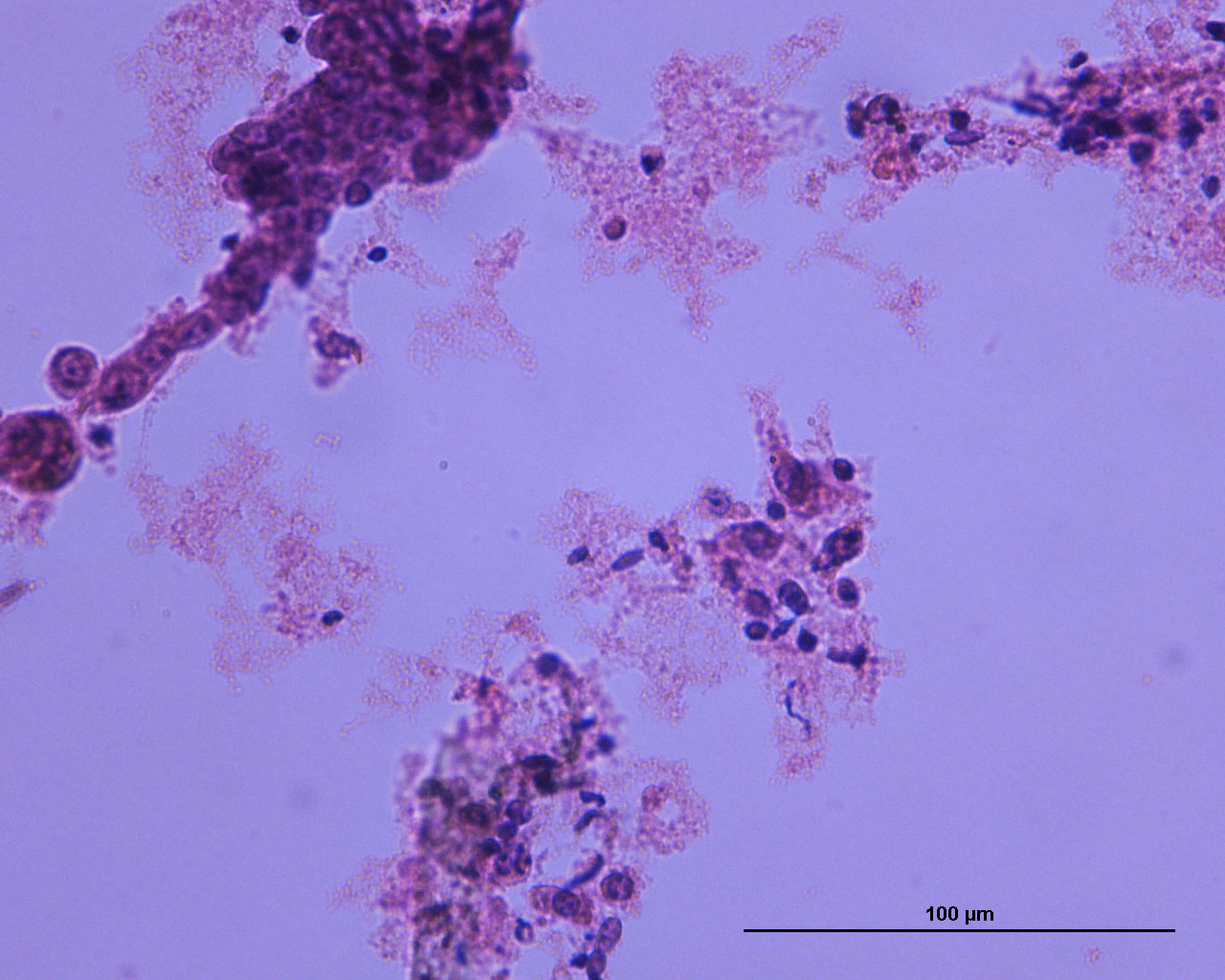

Supplement: S21 Fig — (JPG) [file pone.0190851.s021.jpg]

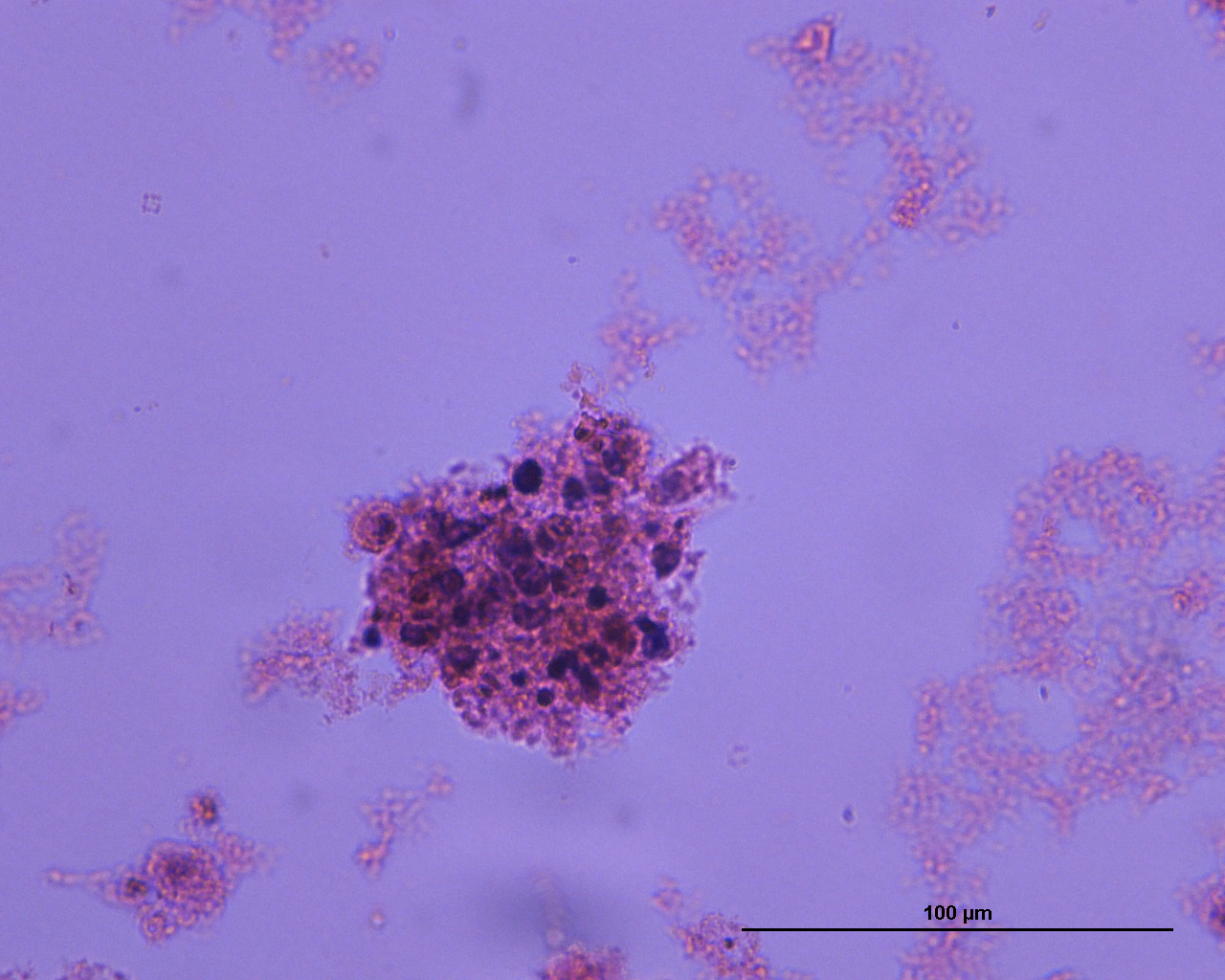

Supplement: S22 Fig — (JPG) [file pone.0190851.s022.jpg]

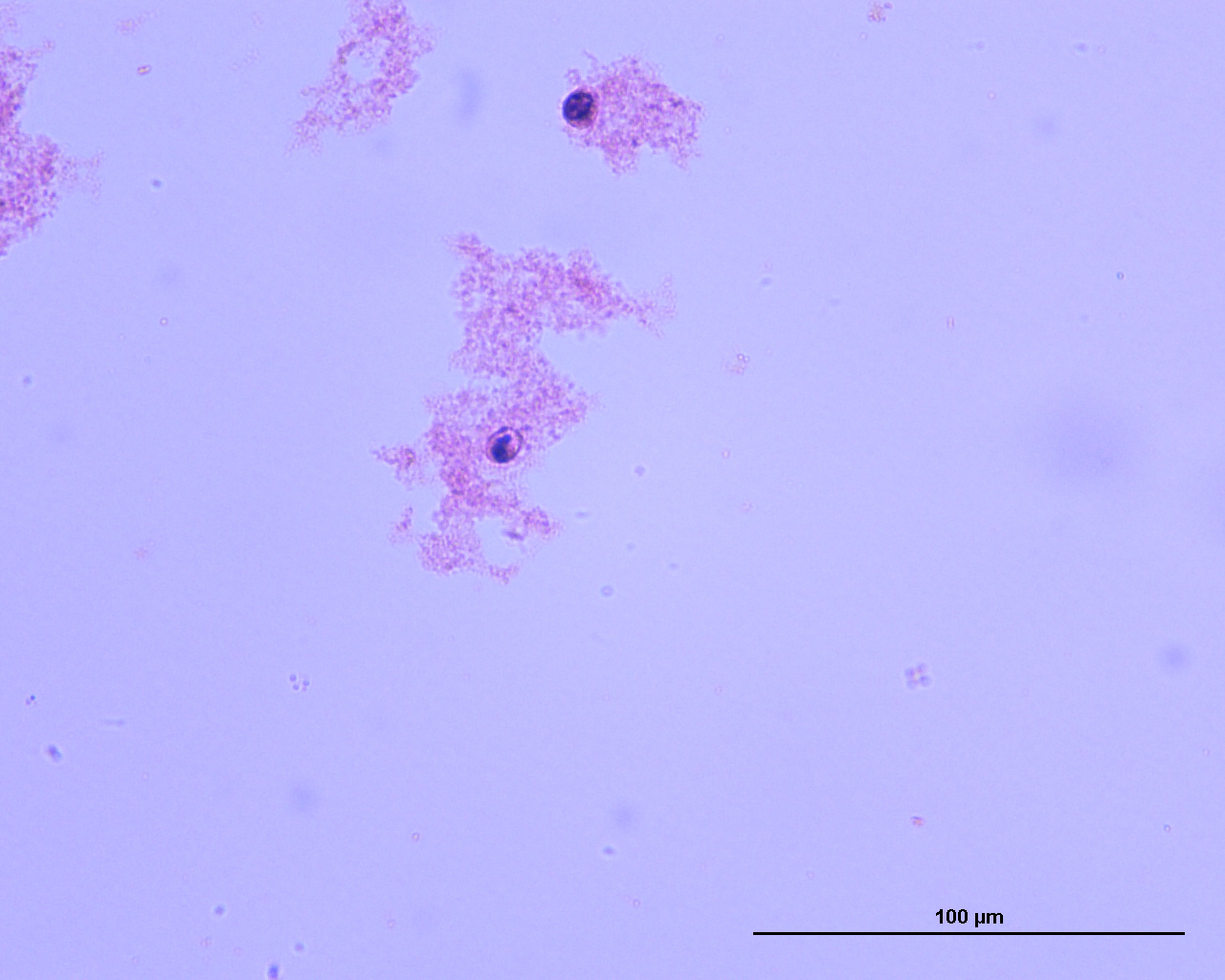

Supplement: S23 Fig — (JPG) [file pone.0190851.s023.jpg]

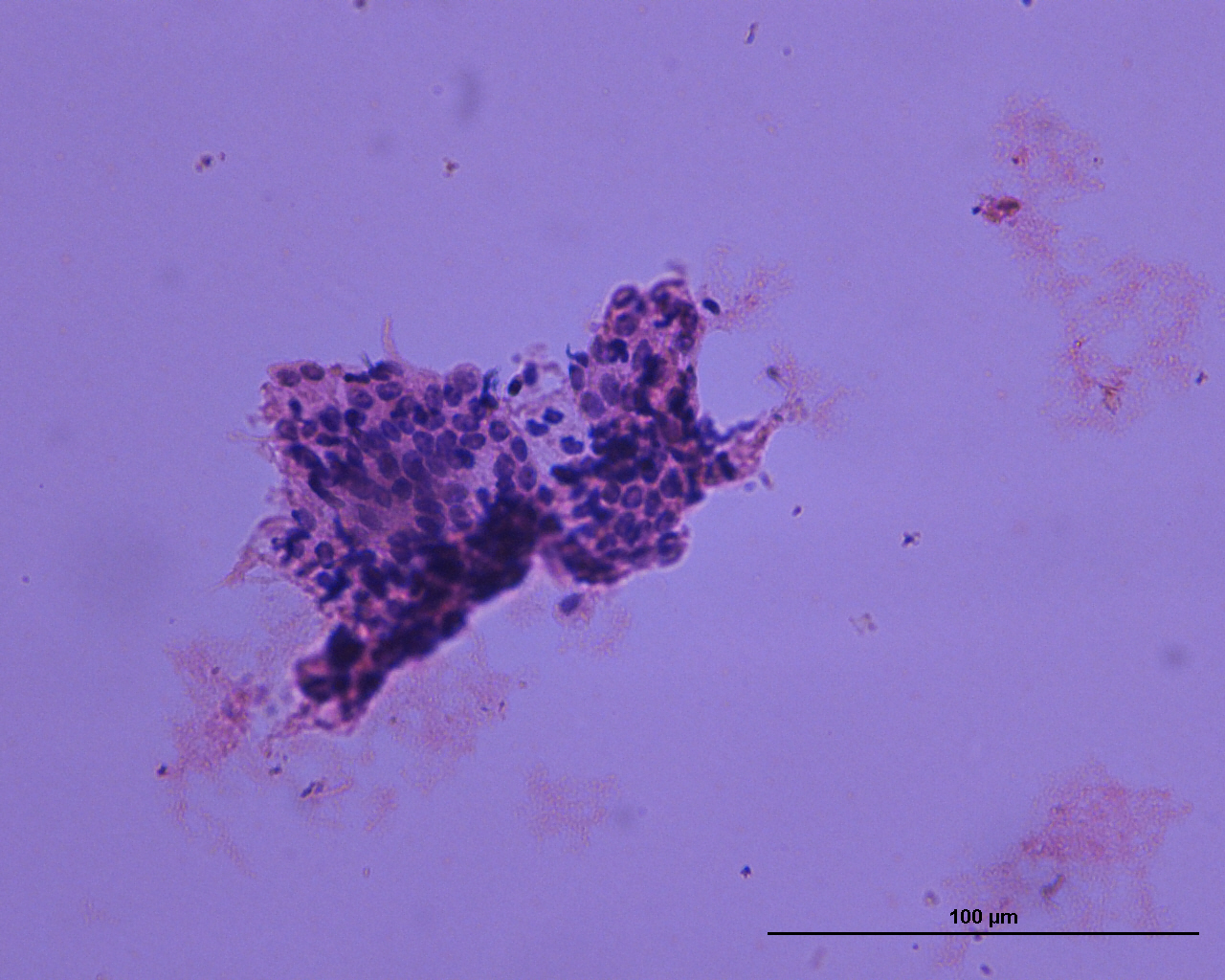

Supplement: S24 Fig — (JPG) [file pone.0190851.s024.jpg]

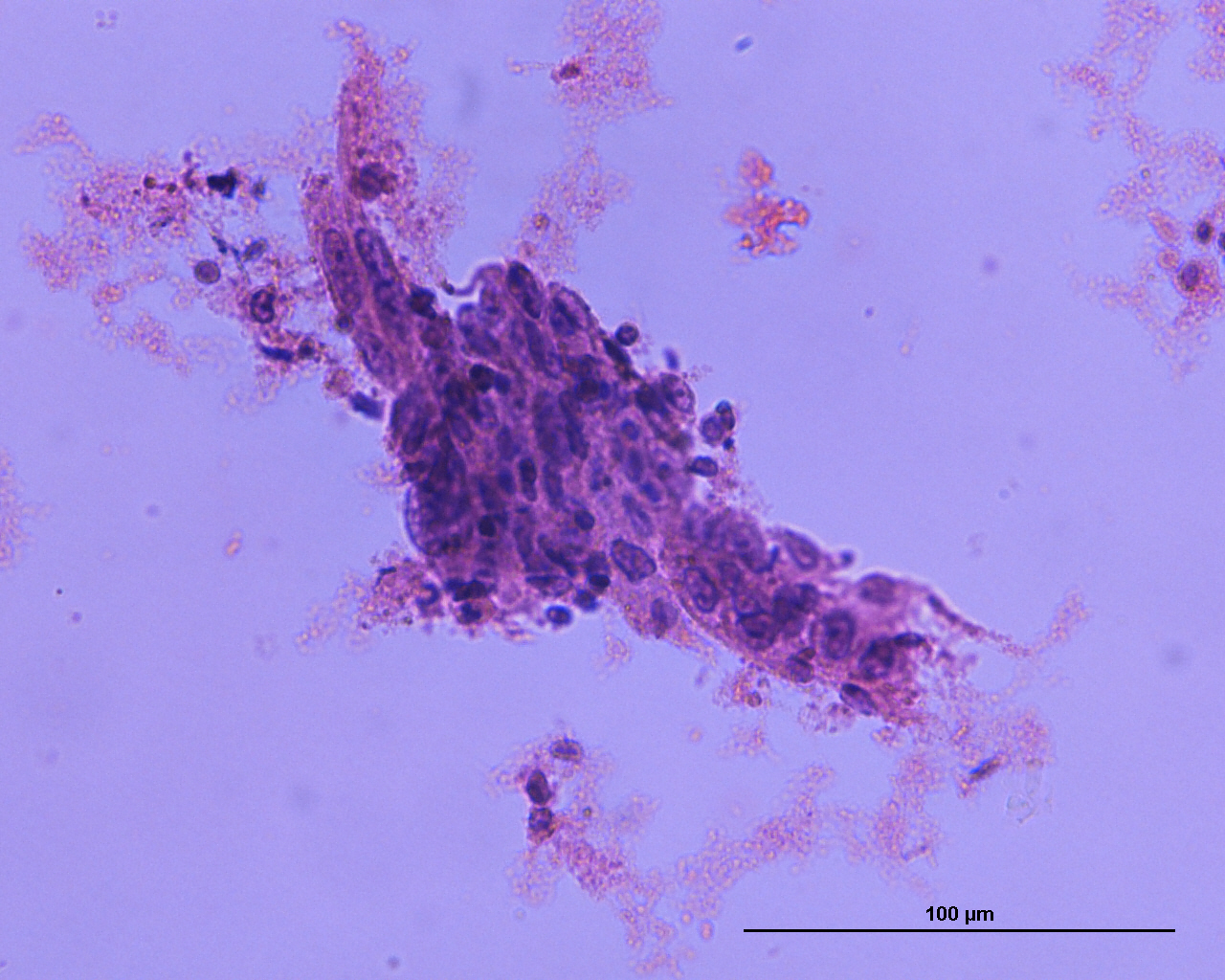

Supplement: S25 Fig — (JPG) [file pone.0190851.s025.jpg]

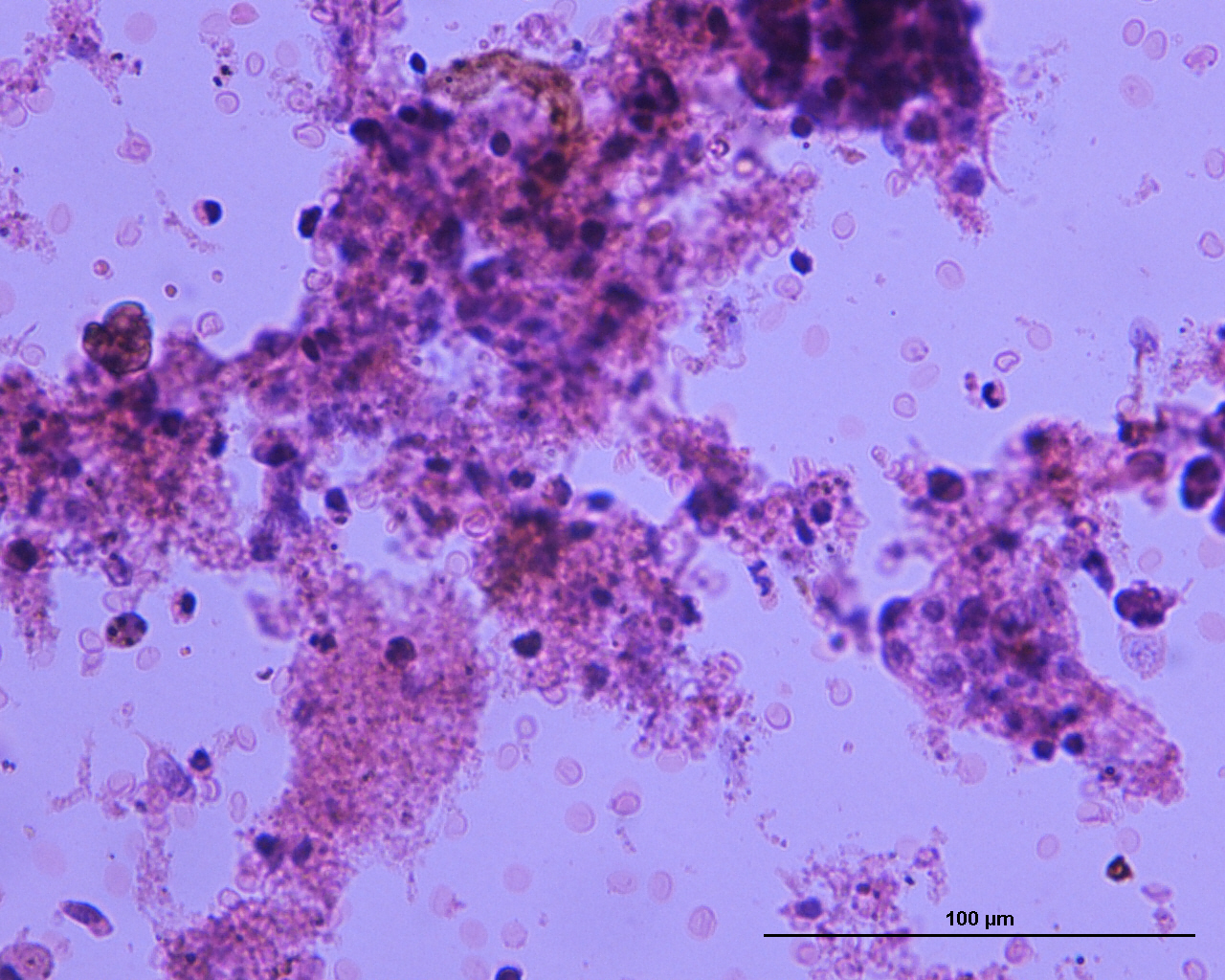

Supplement: S26 Fig — (JPG) [file pone.0190851.s026.jpg]

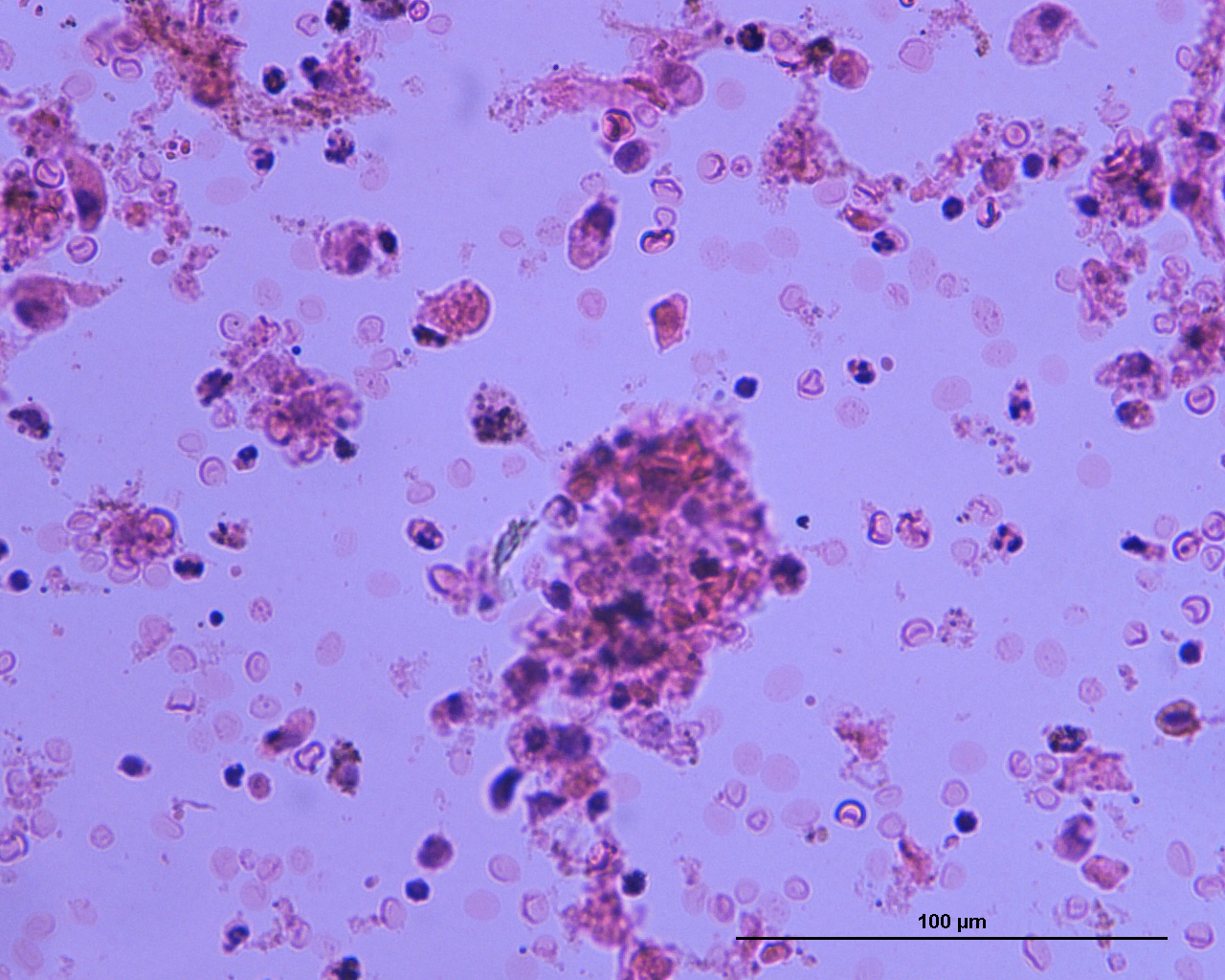

Supplement: S27 Fig — (JPG) [file pone.0190851.s027.jpg]
